# A caddisfly larva case–mimicked gel polymer electrolyte with high toughness and enhanced ion transport for safe lithium metal batteries

**DOI:** 10.1126/sciadv.aed3988

**Published:** 2026-07-08

**Authors:** Quan Liu, Xin Liang, Bing Liu, Yuan Yuan, Kang Wang, Yuxin Xia, Bingbing Li, Huakun Liu, Shixue Dou, Xiang Li, Huaxia Deng, Xinglong Gong

**Affiliations:** ^1^ CAS Key Laboratory of Mechanical Behavior and Design of Materials, Department of Modern Mechanics, University of Science and Technology of China, Hefei 230027, Anhui, PR China.; ^2^ Advanced Institute for Ocean Research, Southern University of Science and Technology, Shenzhen 518055, PR China.; ^3^ School of Energy Materials and Chemical Engineering, Hefei University, Hefei 230000, PR China.; ^4^ Anhui Weiwei Rubber Part Group Co., Ltd., Tongcheng 231460, Anhui, PR China.; ^5^ Institute of Energy Materials Science (IEMS), University of Shanghai for Science and Technology, Shanghai 200093, PR China.; ^6^ Institute for Superconducting and Electronic Materials (ISEM), Australian Institute for Innovative Materials (AIIM), University of Wollongong, Wollongong, NSW 2500, Australia.; ^7^ State Key Laboratory of Nonlinear Mechanics, Institute of Mechanics, Chinese Academy of Science, 15 Beisihuan West Road, Beijing 100190, PR China.; ^8^ State Key Laboratory of Fire Science, University of Science and Technology of China, 96 Jinzhai Road, Hefei 230026, Anhui, PR China.

## Abstract

Lithium (Li) metal batteries (LMBs) promise higher energy density than Li-ion cells but face a trade-off between ionic conductivity and mechanical strength in gel polymer electrolytes (GPEs). Inspired by caddisfly larvae cases, which assemble silk with particles for toughness and permeability, we develop a caddisfly larva case–mimicked gel polymer electrolyte (CLC GPE) with high toughness and enhanced ion transport. It integrates an electrospun polyvinylidene fluoride-hexafluoropropylene scaffold with shear thickening fluid. CLC GPE achieves a high ionic conductivity (2.80 × 10^−3^ siemens per centimeter), a Li^+^ transference number of 0.89, superior toughness (7.29 megajoules per cubic meter), and a puncture energy of 49.69 millijoules, which can resist thermal abuse (150°C), flame, and bullet impact (225 kilometers per hour). Symmetric Li||Li cells exhibit stable cycling over 800 hours, while Li||LFP (LiFePO_4_) full cells maintain 97.6% capacity after 700 cycles at 0.5C. This design couples mechanical robustness with fast ion transport, offering a scalable route to safer, high-performance LMBs.

## Teaser

A caddisfly larva case–mimicked gel polymer electrolyte combines high ionic conductivity with enhanced safety features.

## INTRODUCTION

Lithium (Li) metal batteries (LMBs) are regarded as the ultimate choice for next-generation high-energy-density storage systems because of the ultrahigh theoretical specific capacity (3860 mA·hour g^−1^) and extremely low electrochemical potential (−3.04 V versus the standard hydrogen electrode) of the Li metal anode (*1*–*4*), thus attracting extensive substantial research interest. As the most widely used electrolyte in current Li batteries, liquid electrolytes (LEs) pose inherent safety risks of liquid leakage and flammability (*5*–*8*). Moreover, they are ineffective in suppressing Li dendrite growth during cycling, which leads to the internal short circuits (*9*). While solid-state electrolytes offer superior thermal stability and mechanical strength properties for improving the safety performance, their practical implementation is hindered by low ionic conductivity and unsatisfactory interfacial compatibility (*10*–*13*).

**Fig. 1.**
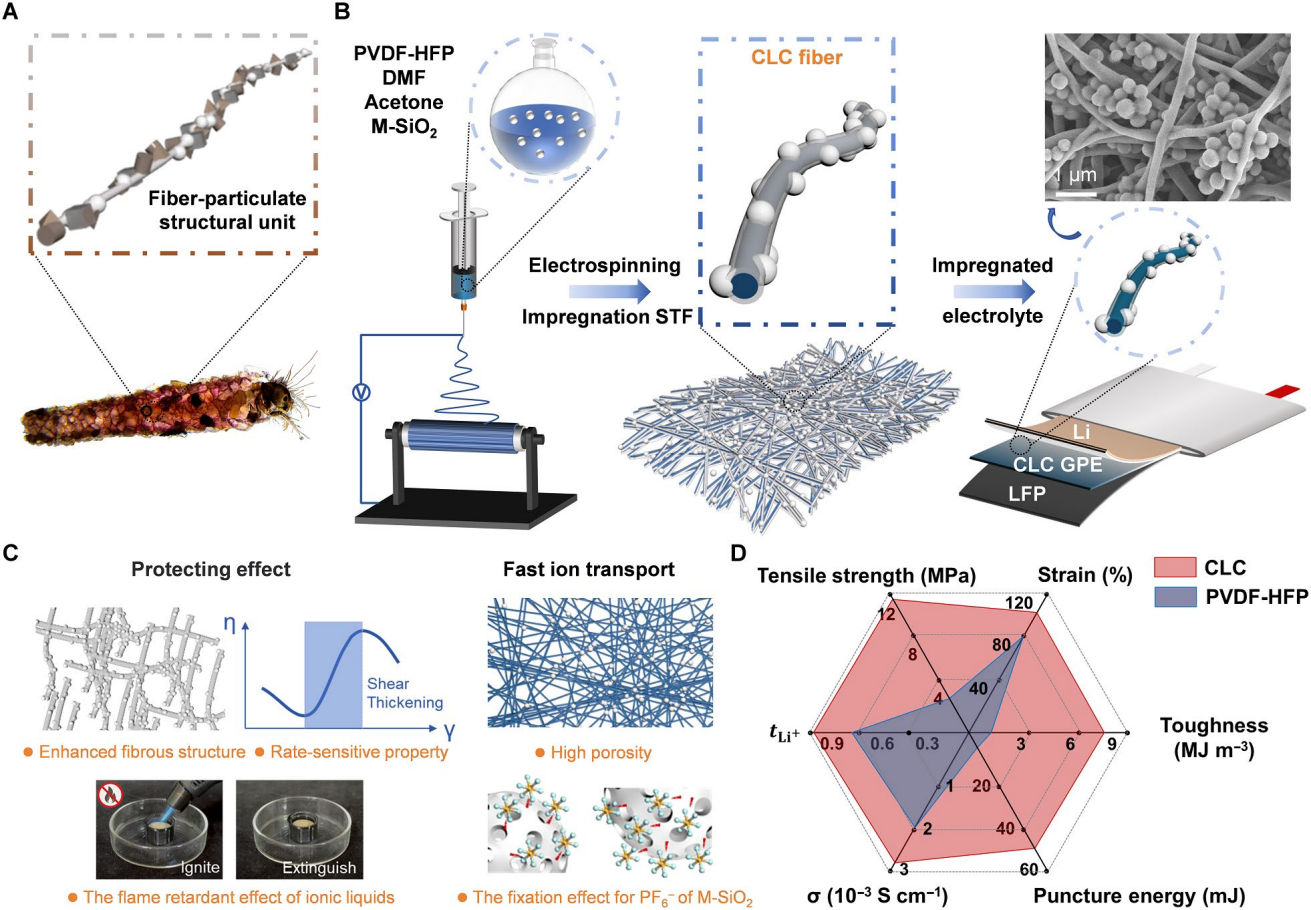
Preparation, design strategies, and performance comparison of CLC GPE. (**A**) Optical images of CLC and schematic diagrams of case. Photo credit: Janeklass, Wikimedia Commons, CCBY-SA 4.0, https://creativecommons.org/licenses/by-sa/4.0/. (**B**) Schematic of the preparation process and structure of CLC GPE. (**C**) Design strategies for CLC GPE with high safety and enhanced ion transport behavior. (**D**) Performance comparison between PVDF-HFP and CLC.

Gel polymer electrolytes (GPEs) demonstrate considerable application potential by combining the great processability and flexibility of polymers with the high ionic conductivity of LEs (*14*–*17*). Striking an optimal balance between high ionic conductivity and desirable mechanical performance in GPEs poses a substantial challenge. For instance, the in situ–polymerized or cross-linked GPEs exhibit robust mechanical strength, while they suffer from relatively low ionic conductivity (typically below 10^−4^ S cm^−1^ at room temperature) because of their dense cross-linked structure and insufficient ion transport pathways (*18*–*20*). Conversely, GPEs derived from electrospun membranes benefit from high porosity and interconnected pore structures, which facilitate rapid ion transport and yield high ionic conductivity (*21*–*27*). Nevertheless, this high porosity often comes at the cost of mechanical strength, leading to the intrinsic drawback of poor mechanical properties (low strength and insufficient toughness) in electrospun membranes (*28*–*32*). The weakness easily leads to the deformation or damage during cell assembly and long-term cycling, introducing potential safety hazards. Consequently, synergistically enhancing the mechanical property and ionic conductivity of GPEs to achieve both high safety and excellent electrochemical performance has become a critical scientific problem demanding urgent solution. In nature, the case built by caddisfly larvae represents a structure that combines rigidity and flexibility with dynamic responsiveness. The caddisfly larval case, architectured by binding environmental particulates with secreted silk fibers, achieves substantial structural robustness while maintaining permeability to both air and water (Fig. 1A). This fiber-particulate composite structure maintains flexibility under normal conditions yet exhibits localized rigidity upon impact through mechanisms such as mechanical interlocking and friction between particles and fibers, enabling effective energy dissipation.

Herein, we report a caddisfly larva case–mimicked GPE (CLC GPE) with high ionic conductivity and enhanced mechanical properties. The electrospun polyvinylidene fluoride-hexafluoropropylene (PVDF-HFP) nanofibrous network mimics the three-dimensional porous proteinaceous scaffold of a caddisfly case, while the infiltrated shear thickening fluid (STF) serves as sand grains that adhere and fill within the fibrous matrix. This unique particle-fiber dual-network structure achieves enhanced mechanical performance of the system, which benefits from the high-strength skeleton formed by the interweaving of particles and fibers as well as the improved structural uniformity of the overall fibrous network. Therefore, the CLC membrane demonstrates a toughness of 7.29 MJ m^−3^, which is higher than that of the pristine PVDF-HFP (1.27 MJ m^−3^). The puncture energy of the CLC membrane reached 49.69 mJ, which is 7.5 times higher than that of the pristine PVDF-HFP membrane. A single-layer pouch cell assembled with CLC GPE survived an impact at 225.0 km/hour without voltage drop and maintained normal charge/discharge operation. Moreover, the introduced ionic liquids (ILs) endow the CLC membrane with flame retardancy, improving the safety of LMBs. Owing to the immobilization of PF_6_^−^ anions by mesoporous silicon dioxide (M-SiO_2_) and the highly porous structure of the fibrous membrane, the CLC GPE exhibited a high ionic conductivity of 2.80 × 10^−3^ S cm^−1^ and a high Li^+^ transference number of 0.89. A Li||CLC GPE||Li symmetric cell demonstrated long-term cycling stability over 800 hours at 0.5 mA cm^−2^ and 0.25 mA·hour cm^−2^. Furthermore, the Li||CLC GPE||LFP cell maintained a high capacity retention of 97.6% after 700 cycles at 0.5C and delivered a high rate capacity of 127 mA·hour g^−1^ at 5C.

## RESULTS

### Design and characterization of the CLC bionic structure

The procedure for preparing the CLC GPE, involving electrospinning, impregnation, and electrolyte soaking, is schematically illustrated in Fig. 1B. The resultant CLC GPE (Fig. 1C) exhibits both the enhanced ion transport behavior and superior safety. The enhanced ion transport behavior primarily arises from the highly porous electrospun fiber architecture and the Lewis acid-base interactions involving M-SiO_2_ (*33*, *34*). M-SiO_2_ effectively immobilizes PF_6_^−^ anions, while its mesoporous structure (fig. S1) facilitates the transport of Li^+^ ions. The enhanced safety is manifested in two aspects: the external impact protection afforded by the shear thickening effect of the STF (fig. S2) and the flame-retardant properties imparted by the incorporated ILs (*35*). Furthermore, the composite CLC membrane demonstrates a reinforced skeleton structure compared with the PVDF-HFP fiber membrane, while the rate-sensitive characteristics of the STF effectively provide resistance against external impacts. As a result, the obtained CLC membrane exhibits superior electrochemical and mechanical properties compared with the PVDF-HFP fiber membrane, achieving a comprehensive performance improvement characterized by an ionic conductivity of 2.80 × 10^−3^ S cm^−1^, a Li^+^ transference number ($t_{{\mathrm{Li}}^{+}}$) of 0.89, a tensile strength of 12.64 MPa, a fracture elongation of 105.26%, a toughness of 7.29 MJ m^−3^, and a puncture energy of 49.69 mJ (Fig. 1D). These properties collectively represent a significant performance enhancement over the unmodified PVDF-HFP fiber membrane. Consequently, this design strategy of CLC GPE holds promise for applications requiring suppression of Li dendrite growth, protection against external impact, and resistance to combustion.

Scanning electron microscopy (SEM) images (fig. S3) reveal that both the electrospun PVDF-HFP and CLC fiber membranes exhibit higher porosity compared with the conventional polypropylene (PP) separator, which is consistent with the measurement results (fig. S4). Furthermore, energy-dispersive spectrometry (EDS) analysis (fig. S5) demonstrates the element distribution of Si and O, indicating a relatively uniform distribution of STF components throughout the membranes. Contact angle measurements (fig. S6) indicate that the CLC membrane has superior electrolyte wettability compared with the PP separator and the PVDF-HFP membrane, thereby facilitating electrolyte infiltration. Consequently, the CLC membrane exhibits a high electrolyte absorption rate of 499% (fig. S7), which is substantially higher than that of PP. The thermal stability of components in the CLC composite was assessed via thermogravimetric analysis (fig. S8). Specifically, the decomposition profile of CLC between 200° and 600°C can be interpreted as the superposition of the decomposition behaviors of the ILs and PVDF-HFP. Notably, the residual mass plateau above 600°C of CLC is notably higher than that of the pristine PVDF-HFP, which is attributed to the presence of residual M-SiO_2_, confirming the successful integration of the STF into the PVDF-HFP polymer matrix. This conclusion is further corroborated by Fourier transform infrared (FTIR) spectroscopy. The spectrum of the CLC composite exhibits characteristic absorption bands associated with the imidazolium cation of the ILs (fig. S9), specifically the N─H stretching vibrations in the range of 3100 to 2500 cm^−1^ and the C═N stretching vibration at 1617 cm^−1^ (*36*, *37*). The mechanical reinforcement imparted by the STF was characterized using rheological testing (fig. S10). Compared with pristine PVDF-HFP, the CLC composite displays a significant enhancement in storage modulus, which is attributed to the reinforcement of the STF on the fibrous membrane’s skeleton. X-ray diffraction (XRD) analysis was used to investigate the influence of STF incorporation on the crystallinity of PVDF-HFP (fig. S11). The XRD pattern of pristine PVDF-HFP exhibits two characteristic peaks at 20.2° and 38.8°, owing to the diffraction from the (110) and (041) crystallographic planes of its typical phase, respectively (*38*, *39*). Upon STF compositing, the diffraction intensity of the (110) plane in CLC is significantly lower than that in pure PVDF-HFP, and the peak corresponding to the (041) plane disappears. These changes indicate a reduction in the overall crystallinity of PVDF-HFP, resulting from the introduction of the elastic STF framework and the nanosized M-SiO_2_ fillers. These structural modifications promote the segmental motion of the polymer chains and consequently enhance Li^+^ ion transport (*40*).

### Electrochemical and mechanical properties

**Fig. 2.**
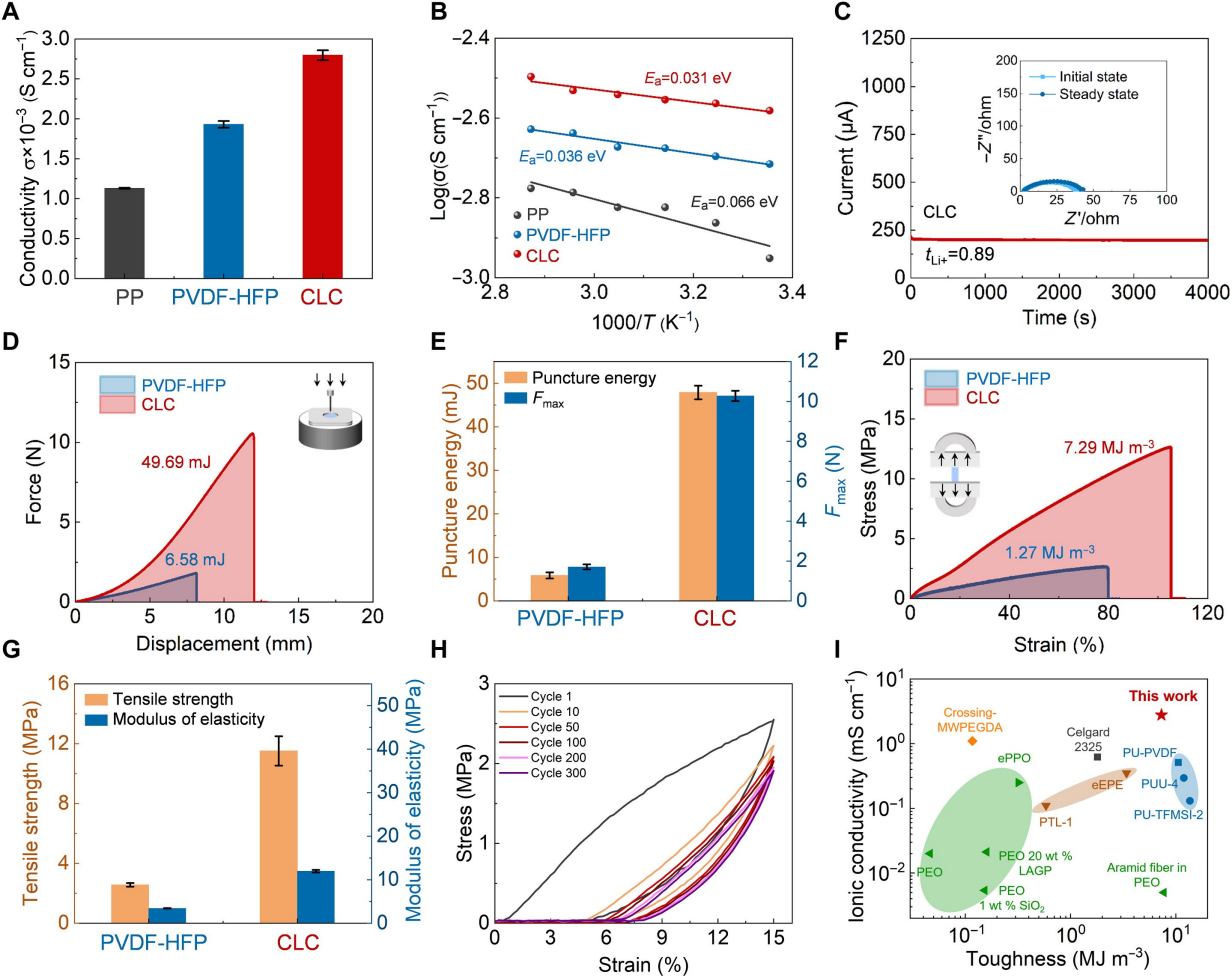
Electrochemical and mechanical properties. (**A**) Ionic conductivities of PP, PVDF-HFP, and CLC GPEs at 25°C. (**B**) Arrhenius plots of different GPEs at various temperatures. (**C**) Li^+^ transference numbers of CLC GPE. (**D**) Puncture force-displacement curves of PVDF-HFP and CLC. The inset shows the schematic of puncture tests. (**E**) Puncture energy and maximum puncture force of PVDF-HFP and CLC. (**F**) Stress-strain curves of PVDF-HFP and CLC. The inset shows the schematic of tensile tests. (**G**) Tensile strength and modulus elasticity of PVDF-HFP and CLC. (**H**) Hysteresis curve of CLC during cyclic stretching at 15% stress. (**I**) Comparison of the toughness and ionic conductivity of CLC with other types of polymer electrolytes. More details are included in table S1.

The electrochemical and mechanical properties of CLC GPE with various PVDF-HFP/STF ratios were investigated. The Nyquist plots and puncture curves of CLC GPE with STF ratios of 0, 15, 25, 35, 45, and 50 wt % are shown in figs. S12 and S13. The ionic conductivity and puncture energy as functions of STF content are summarized in fig. S14. The results demonstrate that CLC GPE with 35 wt % STF exhibits the optimal ionic conductivity and mechanical performance. Therefore, the ratio of 35 wt % was adopted for all subsequent characterizations and measurements. Owing to the highly porous structure of electrospun fiber and Lewis acid-base effect of the M-SiO_2_ filler, the CLC GPE exhibits a high ionic conductivity of 2.80 × 10^−3^ S cm^−1^ (Fig. 2A), which is consistent with its lower bulk resistance (fig. S15). This value is significantly higher than that of the PVDF-HFP GPE (1.93 × 10^−3^ S cm^−1^) and is approximately 2.5 times higher than the conductivity of a conventional PP separator (1.13 × 10^−3^ S cm^−1^). To investigate the activation energy (*E*_a_) for Li^+^ transport within the electrolytes, the bulk resistances of PP, PVDF-HFP GPE, and CLC GPE were measured at various temperatures (fig. S16). The relationship between log(σ) and 1000/*T* was fitted according to the Arrhenius equation (*41*). The CLC GPE exhibits an *E*_a_ of 0.031 eV, which is lower than those of the PVDF-HFP GPE (0.036 eV) and the PP-based electrolyte (0.066 eV) (Fig. 2B). This reduction is attributed to the introduction of M-SiO_2_, which immobilizes PF_6_^−^ anions via Lewis acid-base interactions, thereby facilitating Li^+^ transport. Chronoamperometry curves for Li||Li symmetric cells using different electrolytes and the corresponding impedance changes before and after chronoamperometry testing are presented in Fig. 2C and fig. S17. The Li-ion transference number ($t_{{\text{Li}}^{+}}$) values of PP, PVDF-HFP GPE, and CLC GPE were calculated to be 0.37, 0.65, and 0.89, respectively. Notably, the significantly higher $t_{{\text{Li}}^{+}}$ of CLC GPE compared to those of PP and PVDF-HFP GPE demonstrates accelerated Li^+^ transport kinetics, highlighting the synergistic effect arising from the highly porous fiber structure and the Lewis acid effect of M-SiO_2_. The attenuated total reflectance FTIR spectra of PVDF-HFP GPE and CLC GPE are shown in fig. S18. The peaks at 840 and 560 cm^−1^ correspond to the stretching vibration and bending vibration of PF_6_^−^, respectively. Compared with the PVDF-HFP GPE, the characteristic peaks of PF_6_^−^ in the CLC GPE exhibit a red shift along with peak broadening, indicating an interaction between PF_6_^−^ and M-SiO_2_ (*42*). The Raman spectra in fig. S19 further verify these results, and the PF_6_^−^ peak in CLC GPE shows a tendency to shift toward higher wavelengths (*43*). Further x-ray photoelectron spectroscopy (XPS) analysis was performed on PVDF-HFP GPE and CLC GPE to examine the changes in the binding energy of PF_6_^−^. As shown in fig. S20, which presents the variations in the P 2p and F 1s binding energy peaks for the different electrolytes, distinct positive shifts are observed in both the P 2p peak and F 1s peak of PF_6_^−^ in CLC GPE compared with PVDF-HFP GPE. This confirms the Lewis acid-base interaction between M-SiO_2_ and PF_6_^−^ (*44*).

The CLC composite membrane demonstrates comprehensively superior mechanical properties compared with the pure electrospun PVDF-HFP membrane owing to the reinforcement of the fibrous skeleton by nanoparticles in the STF. The CLC membrane exhibits a puncture-resistant energy of 49.69 mJ (Fig. 2, D and E), which is more than 7.5 times greater than that of the pure PVDF-HFP membrane (6.58 mJ), and a maximum puncture force of 10.57 N. These mechanical properties are crucial for mitigating short circuits and dendrite penetration in LMBs. Tensile testing reveals that the pristine PVDF-HFP membrane exhibits a tensile strength of 2.60 MPa, a fracture elongation of 79.50%, and a toughness of 1.27 MJ m^−3^. The CLC composite membrane achieves significantly enhanced properties: a tensile strength of 12.64 MPa, a fracture elongation of 105.26%, and a toughness of 7.29 MJ m^−3^ (Fig. 2, F and G), representing a substantial overall improvement. When subjected to cyclic tensile loading at 15% strain, the CLC membrane retains 75.5% of its initial maximum stress after 300 cycles, demonstrating its excellent fatigue resistance (Fig. 2H). Furthermore, once the CLC membrane is subjected to 105 cycles under a high tensile stress of 2.5 MPa, the postcycling stress-strain curve shows that the proposed membrane retains a tensile strength of 8.61 MPa (figs. S21 and S22). A comparative analysis of ionic conductivity versus toughness across various reported polymer electrolytes indicates that the CLC GPE strikes a favorable balance between these two crucial properties, surpassing the performance of most reported polymer electrolyte systems (Fig. 2I).

The thermal stability and flame retardancy of the membranes were further evaluated. Thermal stability tests shows that the CLC membrane maintains structural integrity at 150°C, whereas the pristine PVDF-HFP membrane undergoes shrinkage starting at 150°C and the PP separator undergoes significant shrinkage starting at 120°C (fig. S23), demonstrating the superior thermal dimensional stability imparted by STF compositing. In addition, owing to the inherent flame-retardant characteristics of the ILs, the CLC membrane experiences area contraction without sustained combustion under direct flame exposure, whereas both the PP separator and pure PVDF-HFP membrane ignite and burn readily (fig. S24), exhibiting excellent flame retardancy.

### Mechanism analysis of the bionic structural CLC

**Fig. 3.**
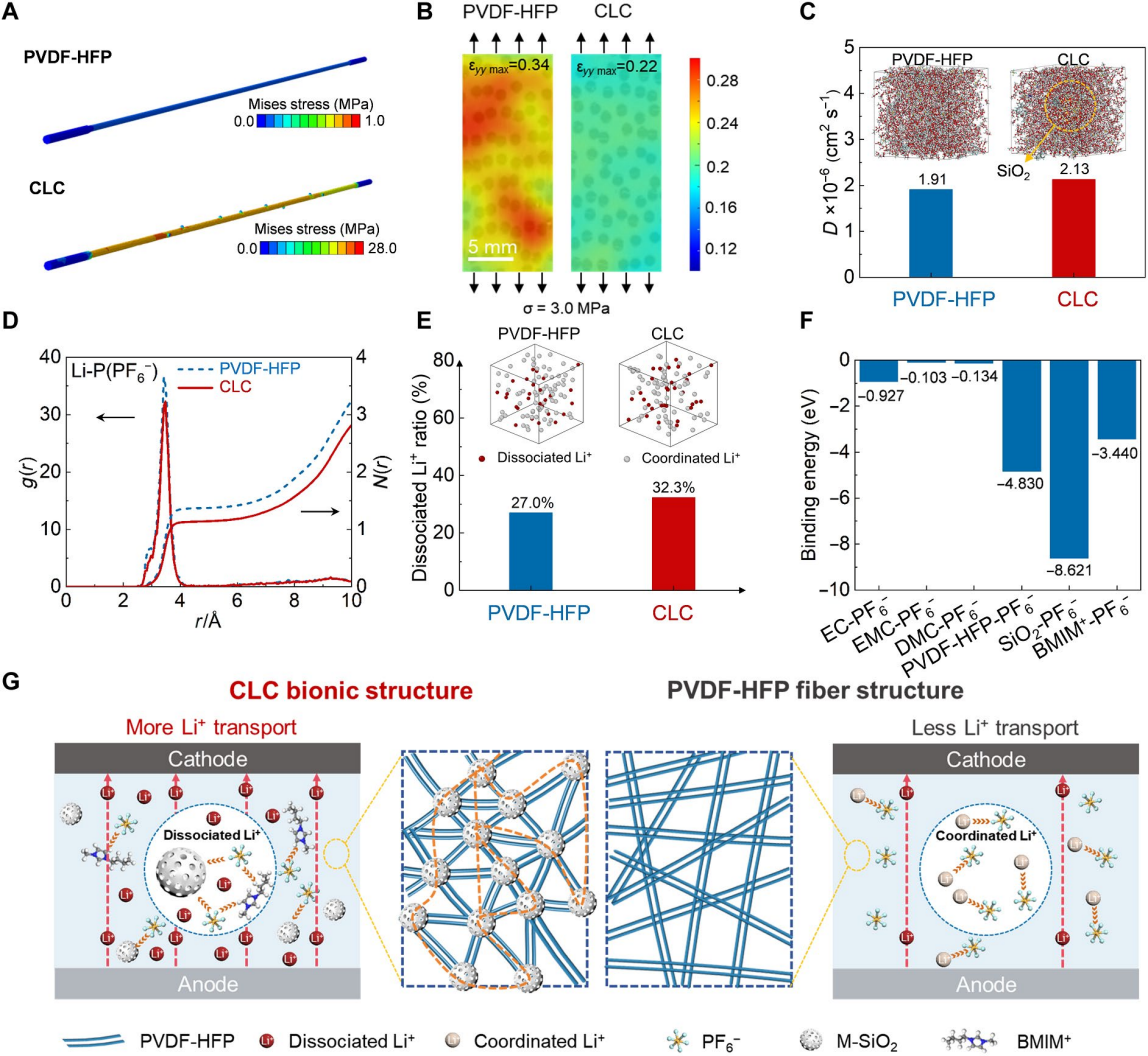
Digital image correlation analysis and the simulation results of the CLC bionic structure. (**A**) Finite element method simulation results for the skeletal structural unit of PVDF-HFP and CLC. (**B**) Strain field ɛ_yy_ of PVDF-HFP and CLC at σ = 3.0 MPa. (**C**) Calculation results of Li^+^ diffusion coefficients in PVDF-HFP and CLC GPE simulation systems. (The insets are the snapshots of MD simulation for PVDF-HFP and CLC GPEs.) (**D**) Corresponding radial distribution function plots and coordination number for two different electrolyte systems collected from MD simulations. (**E**) Proportion of dissociated Li^+^ ions in the MD simulation system for PVDF-HFP and CLC GPEs. (**F**) Binding energies of different interactions in CLC GPE. (**G**) Schematic illustration of the mechanism for CLC GPE achieving high mechanical toughness and high ionic conductivity.

To investigate the mechanical enhancement mechanism of the CLC biomimetic structure, a finite element method model was established to simulate the uniaxial stretching for the skeletal structural unit of PVDF-HFP and CLC. The simulation results (Fig. 3A) demonstrated that the skeletal structural unit of CLC has a higher tensile strength under the same applied strain. Moreover, uniaxial tensile tests were conducted on CLC membranes and PVDF-HFP membranes to further investigate the mechanical enhancement mechanism. An industrial charge-coupled device camera was used to record the morphological evolution of the two types of fibrous membranes during stretching. Noncontact digital image correlation technology was used to analyze their deformation behaviors in-depth under tensile loading. Comparative analysis of strain fields under the same stress conditions (Fig. 3B and fig. S25) reveals that the PVDF-HFP membrane exhibits higher strain values and inhomogeneous strain distribution, which is rooted in the uneven stress transfer caused by internal defects or structural inhomogeneities that lead to local strain concentration. In sharp contrast, the CLC membrane shows significantly reduced strain and uniform strain distribution, demonstrating that its biomimetic structure can effectively regulate deformation behavior. The uniform strain distribution of the CLC membrane is attributed to the effective synergistic effect between the particle phase and the fibrous network: The dispersed particles not only provide structural support but also disperse stress, facilitating uniform transmission and redistribution of external stress in the fibrous network, avoiding excessive local stress concentration, and enabling coordinated and uniform deformation of the entire membrane. Collectively, these results confirm that the “particle-fiber” dual-network biomimetic structure of CLC effectively improves the structural uniformity of the membrane during stretching, which is crucial for enhancing the uniformity of the material’s mechanical properties.

The experimental findings were further validated and elucidated by molecular dynamics (MD) simulations. The calculated Li^+^ diffusion coefficients of the PVDF-HFP GPE and CLC GPE simulation systems indicate that the incorporation of SiO_2_ into the electrolyte matrix has little effect on the Li^+^ diffusion rate (Fig. 3C and fig. S26). Analysis of the radial distribution function revealed Li─P coordination peaks around 3.5 to 4 Å for both the PVDF-HFP GPE and CLC GPE (Fig. 3D). The average coordination number of Li^+^ with PF_6_^−^ in CLC GPE (1.12) was lower than that in PVDF-HFP GPE (1.36). Concurrently, the Li^+^ solvation structure in the CLC GPE system shifted toward higher oxygen coordination (figs. S27 and S28), indicating that M-SiO_2_ and 1-butyl-3-methylimidazole cation (BMIM^+^) can modulate the Li^+^ solvation sheath within the CLC GPE, thereby facilitating rapid Li^+^ transport. As presented in Fig. 3E, the fraction of dissociated Li^+^ in CLC GPE is 32.3%, notably higher than that of the pristine PVDF-HFP GPE (27.0%). The higher proportion of dissociated Li^+^ in CLC GPE confirms that the biomimetic composite framework effectively promotes the dissociation of Li salts, reduces the ratio of solvent/anion–coordinated Li^+^, and provides more movable Li^+^ carriers for efficient ion migration. This quantitative result directly reveals the fundamental mechanism for the significantly enhanced ionic conductivity of CLC GPE and strongly supports the superiority of the proposed biomimetic structural design in synchronously optimizing mechanical and electrochemical properties. The binding energies between different particle pairs within the CLC GPE system were calculated (Fig. 3F). The binding energies between M-SiO_2_ and PF_6_^−^ (−8.621 eV) and BMIM^+^ and PF_6_^−^ (−3.440 eV) are significantly higher than those between solvent molecules and PF_6_^−^, confirming that both M-SiO_2_ and BMIM^+^ effectively immobilize PF_6_^−^ anions in the CLC GPE, consequently promoting Li^+^ cation transport.

As shown in Fig. 3G, the CLC GPE is elaborately designed via a dual-network structure strategy inspired by the structure of caddisfly larvae cases, where rigid particles are interwoven with flexible fibrous networks. This unique construction enables a GPE system with integrated high mechanical toughness and excellent ionic conductivity. The biomimetic “particle-fiber” dual-network structure strategy endows the CLC GPE with a high-strength skeleton and uniform structure. Meanwhile, the Lewis acid effect and high porosity of fibrous networks (94%) facilitate Li salt dissociation and elevates the fraction of dissociated Li^+^, thereby achieving rapid ion transport kinetics.

### Anti-impact safety performance of the CLC GPE pouch cell

**Fig. 4.**
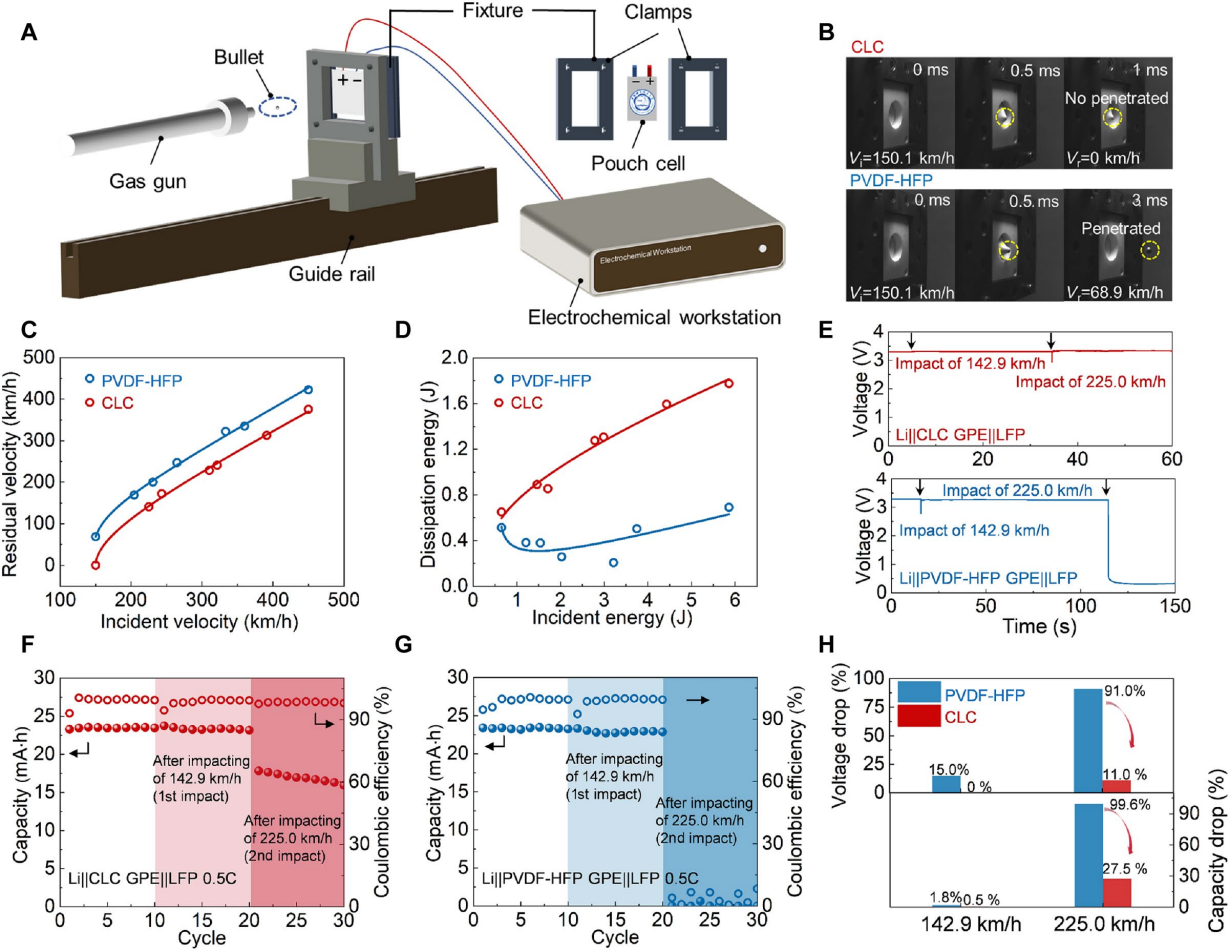
Anti-impact properties of Li||LFP pouch cells with different types of electrolytes. (**A**) Schematic diagram of the ballistic impact test device. (**B**) Snapshots of the bullet impact process of 150.1 km/hour on PVDF-HFP and CLC. h, hour. (**C**) Residual velocity of samples at various incident velocities. (**D**) Energy dissipation of samples at various incident energies. (**E**) Real-time open-circuit voltage curves of pouch cells based on PVDF-HFP and CLC GPEs under bullet impact at different speeds. Cycling performance of (**F**) CLC and (**G**) PVDF-HFP GPE pouch cells before and after bullet impact. (**H**) Comparison of the percentages of voltage drop and capacity drop of PVDF-HFP and CLC GPE pouch cells after two impacts.

Ballistic impact tests coupled with high-speed photography were used to evaluate the anti-impact safety performance of proposed cells (Fig. 4A) and investigate the protective effect of the CLC fibrous membrane against dynamic external impact. Figure 4B and movies S1 and S2 illustrate the impact process of a bullet impacting the PVDF-HFP and CLC membranes at an initial velocity of 150.1 km/hour. The pure PVDF-HFP membrane was easily penetrated, while the CLC membrane remained intact, which is attributed to the skeleton-reinforced fibrous structure and the internal shear thickening effect of STF to dissipate the impact energy. Upon further increasing the bullet velocity, fig. S29 and movies S3 and S4 show that the bullet exhibits a 212.8 km/hour residual velocity after penetrating the CLC membrane. In comparison, the corresponding value of PVDF-HFP is 247.3 km/hour. The residual velocities of the bullet at various impact velocities are plotted in Fig. 4C. The data were fitted using the Recht-Ipson model function (*45*): $v_{r}=\alpha {\left(v_{i}^{p}-v_{b}^{p}\right)}^{\frac{1}{p}}$, where α and p are material-specific constants; $v_{r}$ and $v_{i}$ are the residual and incident velocities of the bullet, respectively; and $v_{b}$ represents the critical impact velocity at which $v_{r}$ becomes 0. The fitting curves clearly indicate that at any given incident impact velocity, the bullet exhibits a consistently lower residual velocity after penetrating the CLC membrane, demonstrating the superior impact resistance imparted by STF compositing. Furthermore, the energy dissipated during the penetration of the two membranes was compared and calculated by using the equation $E_{\text{dis}}=\frac{1}{2}mv_{i}^{2}-\frac{1}{2}mv_{r}^{2}$, where m is the mass of the bullet (Fig. 4D). Significantly more energy was dissipated during the impact with the CLC membrane compared with the pure PVDF-HFP membrane, highlighting the excellent energy dissipation characteristic of the CLC composite.

To further demonstrate the practical impact protection capability of CLC GPE, single-layer Li||LFP pouch cells based on both PVDF-HFP and CLC GPEs were assembled for ballistic impact tests. The open-circuit voltage across the cell terminals was monitored in real time during impact. As shown in Fig. 4E, the pouch cell using CLC GPE exhibited only minor open-circuit voltage fluctuations following two impacts at different velocities. In contrast, the cell with PVDF-HFP GPE showed a slight voltage drop after the first impact (142.9 km/hour) and a sudden drop to 0 V after the second impact (225.0 km/hour), indicating an internal short circuit. The influence of external impact on the cycle life of the batteries was further studied by comparing their postimpact cycling performance (Fig. 4, F and G). The Li||LFP pouch cell with CLC GPE maintained nearly unchanged discharge capacity after the first impact, with only a slight drop following the second impact. By contrast, the cell with PVDF-HFP GPE suffered a significant capacity decline postimpact, suggesting severe internal damage. As summarized in Fig. 4H, the CLC GPE–based cell exhibited markedly smaller percentages of voltage drop and capacity fade after two impacts compared with the PVDF-HFP GPE counterpart, demonstrating the exceptional protective property of CLC GPE under external impact. This underscores its substantial potential for practical application in high-safety secondary battery systems.

The drop hammer collision experiments were further carried out to verify the enhanced safety performance of the CLC GPE (fig. S30). The impact energy on the pouch cell was adjusted by varying the drop height of the hammerhead. The voltage-time profiles of pouch cells using CLC GPE and PVDF-HFP GPE under different impact energies are shown in fig. S31. The cell with CLC GPE showed only slight voltage fluctuations under various impact conditions. Even after a 3.3-J impact, the voltage dropped briefly and then recovered quickly. In contrast, the cell with PVDF-HFP GPE suffered a short circuit at the same impact energy, highlighting the improved safety endowed by the CLC GPE. The nail penetration test on the CLC GPE pouch cell is further studied in fig. S32. During nail penetration, the cell voltage experiences a transient drop followed by rapid recovery, with no occurrence of short-circuiting or electrolyte leakage. In addition, the cell remains capable of powering a light-emitting diode (LED) lamp after penetration, highlighting its outstanding safety performance. Further compression testing revealed that the pouch cell based on CLC GPE showed no obvious voltage drop under a compressive force of 4500 N, and there was no rupture or electrolyte leakage, which further confirms its safety performance (fig. S33).

### Inhibit dendrite performance of CLC GPE

**Fig. 5.**
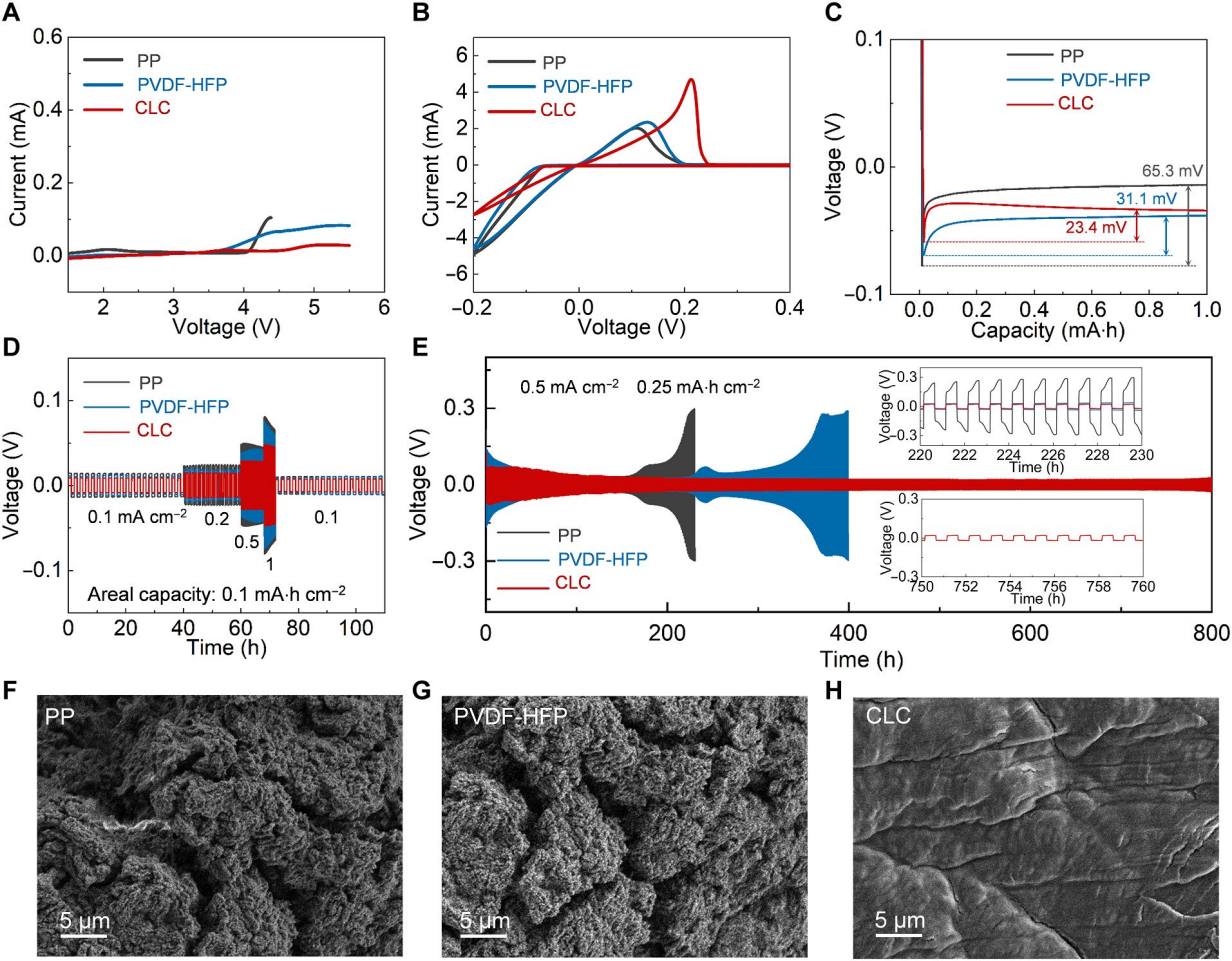
Li plating/stripping behaviors of CLC GPE. (**A**) LSV curves of Li||steel cells in different GPEs. (**B**) CV curves at 1 mV s^−1^ of Li||Cu cells with different GPEs. (**C**) Li nucleation overpotentials on the surface of Cu foil in different GPEs tested by assembling Li||Cu cells. (**D**) Voltage-time curves of Li||Li symmetric cells under different current densities. (**E**) Long-term Li plating/stripping voltage curves of the CLC GPE compared with PVDF-HFP GPE and PP at 0.5 mA cm^−2^ with a fixed capacity of 0.25 mA·hour cm^−2^. SEM images of Li anodes after cycling from (**F**) Li||PP||Li, (**G**) Li||PVDF-HFP GPE||Li, and (**H**) Li||CLC GPE||Li cells.

A series of electrochemical performance tests were conducted on assembled Li symmetric cells to evaluate the influence of CLC GPE on the Li anode. Linear sweep voltammetry (LSV) was performed on Li||steel cells with different electrolytes to evaluate their electrochemical stability windows. The results show that the CLC GPE exhibited no significant oxidation current below 4.5 V. In contrast, distinct oxidative decomposition occurred for both the PVDF-HFP GPE and PP separator above 3.6 and 4.0 V, indicating the superior stability and oxidation resistance of the CLC GPE (Fig. 5A). Typical cyclic voltammetry (CV) curves of Li||Cu cells with different GPEs are shown in Fig. 5B, where the reduction and oxidation peaks correspond to the plating and stripping processes, respectively. The CV curve for the CLC GPE exhibits the largest integrated area, indicating enhanced redox reaction kinetics. The Li nucleation overpotential on the Cu foil substrate was investigated by assembling Li||Cu cells with different GPEs. The results revealed a nucleation overpotential of 23.4 mV for the CLC GPE, which is lower than those for the PVDF-HFP GPE (31.1 mV) and the PP separator (65.3 mV) (Fig. 5C). The lowest nucleation barrier within the CLC GPE can effectively regulate the behavior of Li deposition.

Electrochemical impedance spectroscopy (EIS) was used to evaluate the interfacial resistance of Li||Li symmetric cells with different GPEs. The corresponding Nyquist plots and an appropriate equivalent circuit model are displayed in fig. S34. The Li||CLC GPE||Li cell exhibited the lowest interfacial charge transfer resistance compared with cells with PP and PVDF-HFP GPE, indicating the fastest Li^+^ transport kinetics (*46*, *47*). The rate capability of Li||Li symmetric cells using different GPEs was investigated at various current densities (Fig. 5D). The Li||CLC GPE||Li cell exhibited a stable voltage hysteresis of only 14 mV at 0.1 mA cm^−1^, which is considerably smaller than that of other types of cells. The results demonstrate the superior efficacy of CLC GPE in suppressing Li dendrite growth compared with both the PP separator and PVDF-HFP GPE. At higher current densities of 0.5 and 1.0 mA cm^−1^, cells with PVDF-HFP GPE and PP showed a pronounced increase in voltage hysteresis, indicating that the intensified polarization resulted from Li dendrite formation. More critically, the Li||PP||Li cell exhibited a significant voltage drop after cycling at 1.0 mA cm^−1^, indicating its internal short circuit, which is attributed to the irregular Li deposition resulting from an inhomogeneous ion transport network and the low ionic conductivity of the PP separator. The long-term cycling stability of Li||Li cells with different GPEs was evaluated at a current density of 0.5 mA cm^−2^ and a fixed areal capacity of 0.25 mA·hour cm^−2^ (Fig. 5E). The Li||CLC GPE||Li cell demonstrated a stable and flat voltage profile throughout 800 hours of operation. By contrast, cells with PVDF-HFP GPE and PP exhibited discernible polarization phenomena after ~230 and 400 hours, respectively. Additional cycling performance at a higher current density of 1 mA cm^−2^ and a capacity of 0.5 mA·hour cm^−2^ is presented in fig. S35. The cell using CLC GPE maintained a low voltage hysteresis and significantly enhanced cycling stability under these conditions, indicating the homogeneous Li deposition. The surface morphologies of the Li anodes at different SEM magnifications after 350 cycles at 0.5 mA cm^−2^ and 0.25 mA·hour cm^−2^ are shown in Fig. 5 (F to H) and fig. S36. The cycled Li metal anodes of cells with PP and PVDF-HFP GPE displayed numerous irregular Li dendrites and substantial volume expansion. In sharp contrast, the Li anode from the cell with CLC GPE exhibited a relatively dense and smooth morphology. Thus, the CLC GPE effectively stabilizes the electrode-electrolyte interface, mitigates polarization, and successfully suppresses Li dendrite growth.

### Electrochemical performance of full cells with CLC GPE

**Fig. 6.**
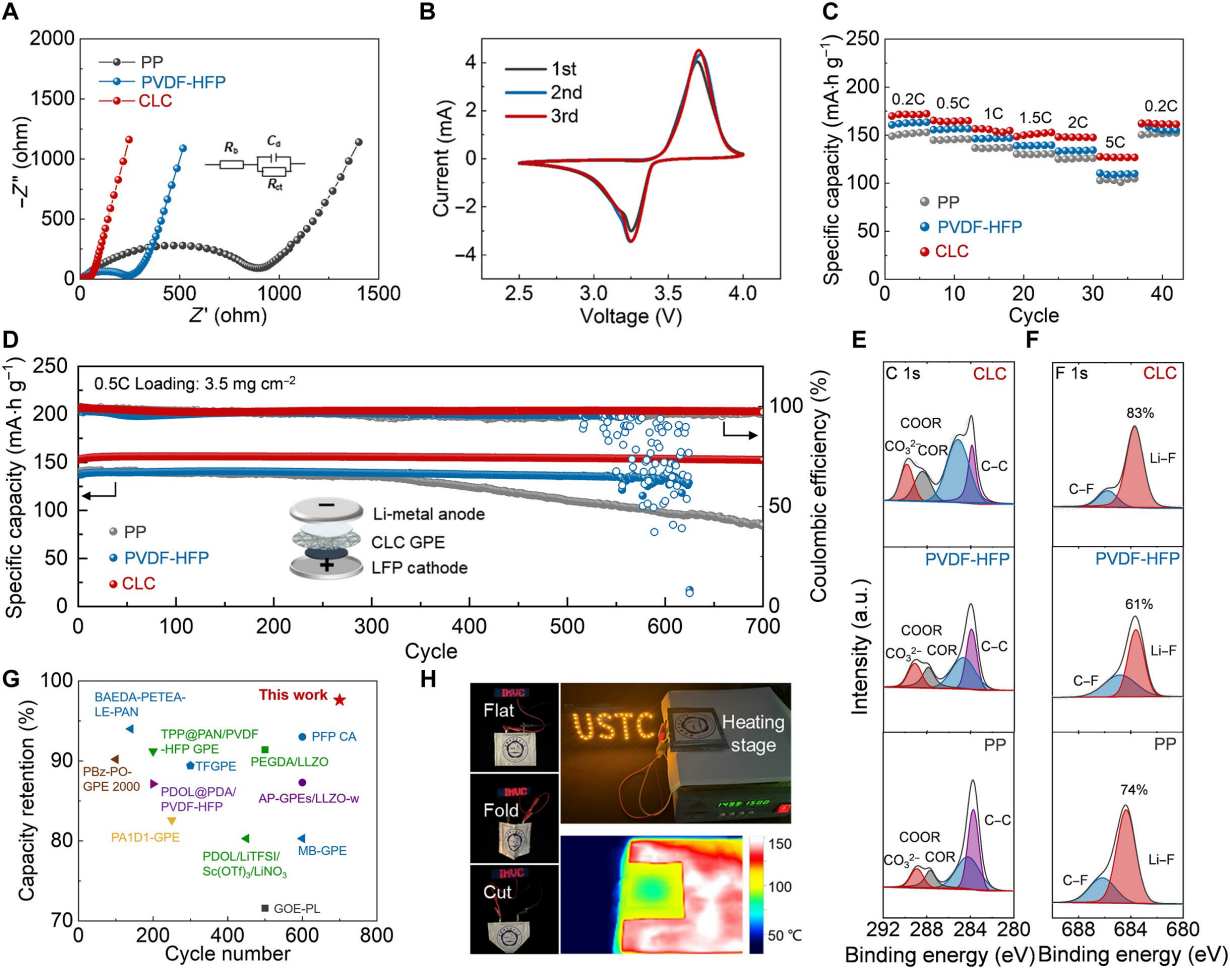
Full-cell performance of the CLC GPE–based cells. (**A**) Nyquist plots and equivalent circuit diagram of full cells. (**B**) CV curves of Li||CLC GPE||LFP cells. (**C**) Rate performances of Li||LFP cells of PP, PVDF-HFP, and CLC GPEs. (**D**) Long-term cycling performance at 0.5C of Li||LFP cells of different GPEs. XPS spectra of (**E**) C 1s and (**F**) F 1s for anodes retrieved from PP, PVDF-HFP, and CLC GPEs after 50 cycles. a.u., arbitrary units. (**G**) Comparison of CLC GPE with the GPEs reported recently in the literature. More details are included in table S2. (**H**) Digital photos of the CLC GPE pouch cell undergoing flatting, folding, and cutting and the infrared thermography of the pouch cell with CLC GPE at 150°C.

Li||LFP full cells were assembled, and their electrochemical performance was characterized to further evaluate the applicability of GPEs and their long-term cycling stability. EIS was used to analyze the internal resistance of the full cells with different GPEs. As shown in Fig. 6A, the Li||CLC GPE||LFP cell exhibited the lowest interfacial charge transfer resistance, which originates from the rapid Li^+^ migration kinetics within the CLC electrolyte. The consistency of CV curves for the Li||CLC GPE||LFP cell is shown in Fig. 6B, indicating highly reversible redox reactions. As shown in Fig. 6C, the Li||CLC GPE||LFP cell exhibited specific discharge capacities of 169, 165, 155, 150, 148, and 127 mA·hour g^−1^ at current densities of 0.2C, 0.5C, 1C, 1.5C, 2C, and 5C, respectively (1C = 170 mA g^−1^). These values are markedly higher than those of full cells based on PP and PVDF-HFP GPE. Notably, the Li||CLC GPE||LFP cell maintained a specific discharge capacity of 127 mA·hour g^−1^ even at a high rate of 5C. Furthermore, the capacity recovered quickly when the current density was returned to 0.2C, demonstrating excellent electrochemical reversibility. The charge-discharge profiles of the full cells with different GPEs at various current densities are presented in fig. S37, showing that the CLC GPE cell exhibits the smallest polarization. In addition, the Li||CLC GPE||LFP cell exhibited a high capacity retention rate of 97.6% after 700 cycles at 0.5C (Fig. 6D and fig. S38), which demonstrates its superior performance than cells with PVDF-HFP GPE and PP. This superior cycling stability is attributed to the enhanced ionic conductivity and improved mechanical strength of the CLC GPE.

To clarify the role of CLC GPE in enhancing LMBs’ cycling stability, XPS was used to analyze the solid electrolyte interphase (SEI) layer composition on Li anodes from LFP||PP||Li, LFP||PVDF-HFP GPE||Li, and LFP||CLC GPE||Li cells before and after 50 cycles at 0.5C. As shown in fig. S39 (C 1s spectra), before cycling, all three systems exhibited organic-dominated initial SEI layers (mainly C─C, COR, and COOR). This indicates that the initial SEI was loose and unstable. After 50 cycles, the CLC GPE group showed significantly stronger Li_2_CO_3_ peaks, confirming a denser organic-inorganic hybrid SEI that suppresses side reactions and stabilizes Li deposition/stripping (Fig. 6E). The SEI-fluorinated components were further illustrated in fig. S39 (F 1s spectra). Weak lithium fluoride (LiF) signals were detected in all three systems before cycling. After 50 cycles, distinct LiF characteristic peaks were detected in all three electrolytes, demonstrating that a stable inorganic framework is formed in the SEI of each system (Fig. 6F). Notably, the LiF peak intensity of CLC GPE is significantly higher than those of the other two, suggesting a higher LiF content and a denser inorganic layer in its SEI film. This can effectively reduce interfacial impedance, suppress Li dendrite growth, and mitigate continuous electrolyte decomposition, thereby enhancing the long-cycle stability of the batteries. To further clarify the composition and crystalline characteristics of the interfacial layer, the crystalline components of the SEI film on the Li anode surface after cycling in three systems were compared through XRD characterization. Compared with the PP and PVDF-HFP GPE groups, the CLC GPE–modified system shows stronger characteristic diffraction peaks of LiF and Li_2_CO_3_ (fig. S40). The results confirm that the CLC GPE can induce the formation of a dense SEI film rich in inorganic crystalline phases, effectively stabilize the Li anode interface, inhibit side reactions and dendrite growth, and provide interfacial support for the excellent long-cycling performance of the battery.

Furthermore, the Li||CLC GPE||LFP cell retained 89% of its initial capacity after 1000 cycles at 2C (figs. S41 and S42), demonstrating exceptional long-term cycling stability. As summarized in Fig. 6G, the performance of our CLC GPE surpasses that of many GPE-based cells reported in the literature. To demonstrate the potential for the application of CLC GPE, pouch cells based on CLC GPE were fabricated. The cycling performance of pouch cells assembled with the CLC GPE under a high LFP cathode loading of 13.53 mg cm^−2^ is presented in fig. S43. At 0.5C, the cell retains a high capacity retention of 91.7% over 300 cycles, with coulombic efficiency consistently above 99.5%. This substantial cycling stability under high-areal-loading conditions validates the great potential of the CLC fibrous membrane for practical large-scale applications. Also, the superior capacity retention confirms that the CLC GPE can sustain stable electrode-electrolyte interfacial contact even under high-areal-capacity working conditions. In addition, the pouch cell maintained its ability to power the LED without causing a short circuit even after being subjected to folding and cutting, demonstrating its outstanding stability and resistance to mechanical deformation (Fig. 6H). The thermal tolerance of the Li||CLC GPE||LFP pouch cell was evaluated by placing it on a hot plate at 150°C (Fig. 6H). Infrared thermography revealed that the cell’s surface temperature remained stable and well below the threshold for thermal runaway. Therefore, the cell successfully powered an LED at this elevated temperature, indicating its superior thermal tolerance.

## DISCUSSION

This work presents a CLC GPE by incorporating an electrospun PVDF-HFP fibrous scaffold with STF. The CLC GPE achieves high ionic conductivity and enhanced mechanical properties simultaneously, including puncture resistance, flame retardancy, and impact tolerance. The CLC membrane demonstrates superior mechanical properties to the pure PVDF-HFP membrane, with a toughness of 7.29 MJ m^−3^ and a puncture energy of 49.69 mJ, which is due to the enhanced tensile strength of CLC individual fibers and the improved structural uniformity of the CLC bionic structure. The CLC membrane withstood the impact loading at an initial velocity of 150.1 km/hour, and pouch cells assembled with this proposed GPE maintained normal operation even under the impact loading velocity of 225 km/hour. Owing to the synergistic effect of the highly porous fibrous framework and Lewis acid-base interaction enabled by M-SiO_2_, the CLC GPE exhibits a high ionic conductivity of 2.80 × 10^−3^ S cm^−1^ and a Li^+^ transference number of 0.89. The symmetric Li||CLC GPE||Li cells showed long-term cycling stability over 800 hours at 0.5 mA cm^−2^ and 0.25 mA·hour cm^−2^. Moreover, the Li||CLC GPE||LFP full cells retained 97.6% capacity after 700 cycles at 0.5C and delivered a high rate capacity of 127 mA·hour g^−1^ at 5C. This work provides a viable strategy for designing highly safe and conductive electrolytes, paving the way for the development of next-generation LMBs.

## MATERIALS AND METHODS

### Materials

Hexadecyltrimethyl-ammonium bromide [CTAB; analytical reagent (AR)], tetraethyl orthosilicate (AR), ammonium hydroxide (NH_3_·H_2_O; AR), ethanol (AR), and hydrochloric acid (AR) were supplied by Sinopharm Chemical Reagent Co., Ltd. ILs were purchased from Aladdin. PVDF-HFP [*M*_w_ (weight-average molecular weight) = 400,000] was purchased from Shanghai Aichun Biological Technology Co., Ltd. *N*,*N*-Dimethylformamide, *N*-methyl-2-pyrrolidone, and acetone were purchased from Sinopharm Chemical Reagent Co., Ltd. Commercial LE of 1 M LiPF_6_ in ethylene carbonate/dimethyl carbonate/ethyl methyl carbonate (EC/DMC/EMC) (volume ratio, ~1:1:1) was provided by Hefei Guoxuan High-Tech Power Energy Co., Ltd. The cathode materials of LFP, conductive agent (Super-P), polyvinylidene difluoride (PVDF), and Li foils (15.6 by 0.45 mm) were purchased from Guangdong Canrd Technology Co., Ltd.

### Preparation process of M-SiO_2_ and STF

M-SiO_2_ nanoparticles were synthesized via a surfactant-templated method. Initially, 3.0 g of CTAB was dissolved in a mixture comprising 450 ml of ethanol and deionized water. Subsequently, 9.8 ml of ethanol was introduced to the solution under vigorous stirring. Following thorough homogenization, 15 ml of tetraethyl orthosilicate was added as the silica precursor. The reaction mixture was then maintained at 45°C with continuous stirring at 600 rpm for a period of 24 hours to facilitate hydrolysis and condensation. The resulting precipitate was isolated by centrifugation and subjected to multiple washing cycles with deionized water and ethanol to remove soluble impurities. To eliminate the templating agent (CTAB), the collected precipitate was dispersed in ethanol, treated with a 5% (v/v) hydrochloric acid solution, and refluxed at 60°C for 4.5 hours. After this acid treatment, the solid product was again recovered by centrifugation and extensively washed with deionized water and ethanol. The washed material was dried in an oven, yielding an intermediate product designated as M-SiO_2_@CTAB. Last, to ensure the complete removal of residual CTAB and achieve the desired mesoporous structure, the M-SiO_2_@CTAB powder was calcined in air at 550°C for 6 hours. A suitable amount of M-SiO_2_ was dispersed into the ball-milling jar filled with IL. The mixture was then homogenized through ball milling. The ball-milling process was carried out in the ball mill for 24 hours, and STF was obtained.

### Preparation process of PVDF-HFP GPE and CLC GPE

The PVDF-HFP fiber membrane was prepared by electrospinning method. PVDF-HFP powder (2.6 g) was dissolved in a mixture of 7 ml of *N*,*N*-dimethylformamide and 3 ml of acetone. After stirring for 6 hours at room temperature, a transparent and uniform spinning solution was obtained. Then, the spinning solution was transferred to an electrospun injector with a high voltage of 15 kV. The solution was spun onto an Al foil at a flow rate of 0.3 ml/hour, with a roller speed of 100 rpm, to obtain the PVDF-HFP fiber membrane with the thickness of 60 μm. To prepare the CLC fiber membrane, 0.26 g of M-SiO_2_ particles was dispersed into the spinning solution to obtain the PVDF-HFP@M-SiO_2_ spinning solution. Through electrospinning, the PVDF-HFP@M-SiO_2_ fiber membrane with the thickness of 60 μm was obtained. The spinning parameters were the same as those used for preparing the PVDF-HFP fiber membrane. The CLC fiber membrane was fabricated via multiple impregnation with ethanol-diluted STF. Dilution with ethanol enables the STF to uniformly infiltrate the fibrous membrane, which is subsequently subjected to oven drying for ethanol evaporation to obtain the CLC fiber membrane. Specifically, the diluted STF was prepared by adding nine times the volume of ethanol to the pristine STF. The PVDF-HFP@M-SiO_2_ fibrous membrane was immersed in the diluted STF for 2 min on both the front and back sides, and this impregnation process was repeated multiple times to ultimately achieve a CLC fiber membrane with an STF impregnation mass fraction of 35 wt %. To obtain the GPE, the fiber membrane was cut into a circular piece with a diameter of 19 mm and then soaked in LE for 24 hours to obtain the GPE. To ensure the uniformity and repeatability of the electrochemical performance, the thickness of the PVDF-HFP GPE and CLC GPE was uniformly set to 60 μm. In addition, the final composition of the CLC GPE is shown in table S3.

### Fabrication of battery cells

Li||Li, Li||Cu, and Li||LFP cells were respectively assembled as CR2032 coin cells using Li foil, Cu foil, and LFP as the anode and cathodes, respectively, with different GPE fiber membranes as the electrolyte, in a glove box with oxygen and water content lower than 0.01 ppm (parts per million). To prepare the LFP cathodes, the cathode slurry was prepared by mixing LFP, conductive agent Super-P and binder PVDF (10 wt %), and solvent *N*-methyl-2-pyrrolidone. The mass ratio of LFP:Super-P:PVDF is 8:1:1. After grinding and mixing evenly, the slurry was scraped onto the current collector aluminum foil using a scraper and then dried overnight at 80°C. Last, the aluminum foil cathode was stamped into a 12-mm-diameter disc. For LFP||Gr pouch cells, a single-sided coated cathode (LFP, with a coating surface density of 13.53 mg cm^−2^, 3.5 cm by 4 cm) and a single-sided coated negative electrode (graphite, with a coating surface density of 5.55 mg cm^−2^, 4 cm by 5 cm) were used as the anode and cathodes of the cells, respectively. For LFP||Li pouch cells, a single-sided coated cathode (LFP, with a coating surface density of 11.25 mg cm^−2^, 3.5 cm by 4 cm) and a Li foil (thickness of 100 μm, 4 cm by 5 cm) were used as the anodes, and different GPE fiber membranes were used as the electrolytes.

### Material characterizations

The morphology of PP, PVDF-HFP, and CLC membranes was characterized by using the field emission scanning electron microscope (GeminiSEM 450). The microstructure of M-SiO_2_ was characterized by transmission electron microscopy (JEM-2100F). The FTIR spectra of different fiber membranes were obtained by using an FTIR spectrometer (Nicolet 8700). The crystallization behavior of the fiber membrane was analyzed by XRD (Super Nova). The thermogravimetric analysis was carried out in a N_2_ (nitrogen gas) atmosphere at a heating rate of 10°C min^−1^ by a thermal gravimetric analyzer (TG 209F1). FTIR, Raman analysis, and XPS were conducted to reveal the Lewis acid-base interaction in CLC GPE and PVDF-HFP GPE. XPS and XRD measurements were performed to analyze the chemical composition and crystalline phases of the SEI film on Li anodes after cycling. The evolution of inorganic and organic SEI components was comparatively investigated to reveal the interfacial regulation mechanism of different GPEs.

### Characterizations on rheological, mechanical, and puncture properties

The rheological properties of STF, PVDF-HFP, and CLC fiber membranes were tested using a rheometer (Anton-Paar MCR 302). The mechanical property of the membranes was measured in a tensile mode with a constant rate of 0.2 mm s^−1^ by Electronic Universal Testing Machine (C43.304E). For the puncture resistance test, the electronic universal testing machine was used to conduct the puncture at a puncture speed of 0.02 mm s^−1^.

### Ballistic test

The impact resistance of PVDF-HFP and CLC fiber membranes was compared through ballistic tests. The thickness of the fiber membranes under the high-speed impact of the bullet was uniformly set at 350 μm. The bullet was a small aluminum ball with a mass of 0.75 g, and the high-speed impact was generated by pneumatic drive. The speed was controlled by the air gun pressure. The 5.5 cm–by–5.5 cm fiber membranes were fixed by a fixture, and the photoelectric gate was used to record the entry speed of the bullet, while high-speed photography recorded the process of the bullet penetrating the fiber membrane and calculated the remaining speed. Impact tests were conducted on pouch cells based on PVDF-HFP GPE and CLC GPE using a ballistic device (the bullet was a small steel ball with a mass of 2.0 g). The impact speed was recorded by photoelectric gates. The impact energy could be obtained by the mass and speed of the bullet. The process of the pouch cell being impacted was recorded in real time by high-speed photography. The open-circuit voltage of the pouch cell was monitored in real time by Chenhua Electrochemical Workstation.

### Electrochemical measurements

All electrochemical tests were carried out in the CR2032-type coin cells. The electrochemical impedance spectra of Li||Li and Li||LFP cells were obtained by using the Chenhua Electrochemical Workstation. The electrochemical window of the Li-stainless steel symmetric cell was tested by LSV. The test was conducted at 25°C with a scan rate of 1 mV s^−1^ within the voltage range of 0 to 5.5 V. Li||Cu cells were assembled with Li foil and Cu foil as the working electrode and reference electrode, respectively, and different GPE films as the electrolytes. The overpotential of Li nucleation was measured by the constant current charge-discharge method. CV curves of Li||Cu cells were tested within the voltage range of −0.2 to 0.4 V at 1 mV s^−1^. CV curves of Li||LFP cells were tested within the voltage range of 2.5 to 4 V at 1 mV s^−1^. The charging and discharging performance of the Li||Li cell was tested under different current densities to investigate the polarization potential generated during the Li platting/stripping process. The cycling performance and rate performance of the Li||LFP cell were obtained by testing on the NEWARE cell testing system, with cycle tests conducted at 0.5C and 2C current densities within a voltage range of 2.5 to 4.2 V. The ionic conductivity σ was calculated by the following equation: $\sigma =\frac{L}{\mathrm{RS}}$, where L represents the thickness of the diaphragm, R represents the bulk resistance obtained through EIS, and S is the cross-sectional area of the electrode. To calculate the activation energy barriers for the interface reactions in different GPE membranes, the charge transfer resistance (*R*_ct_) of the Li||Li symmetric cell was measured at different temperatures (25° to 75°C). The diameter of the semicircle in the high-frequency region represents the charge transfer resistance. On the basis of the Arabius equation, the activation energy ($E_{a}$) can be calculated: $\text{In}k=-\frac{E_{a}}{\mathrm{RT}}+\text{In}A$, where k is the reaction rate constant; *A* is the Arrhenius constant; $E_{a}$ is the activation energy barrier, which can be regarded as a constant within a small temperature range; R is the gas constant (8.314 J mol^−1^ K^−1^); and T is the converted kelvin test temperature (K). Constant potential polarization combined with EIS technology was used to evaluate the Li^+^ migration number ($t_{{\text{Li}}^{+}}$) in Li||Li symmetric cells. $t_{{\text{Li}}^{+}}$ was determined by the following equation: $t_{{\text{Li}}^{+}}=\frac{I_{S}\left(∆V-I_{i}R_{i}\right)}{I_{i}\left(∆V-I_{S}R_{S}\right)}$, where ∆V is the applied voltage (10 mV), $I_{i}$ and $R_{i}$ are the initial current and charge transfer resistance, and $I_{S}$ and $R_{S}$ refer to the steady-state current and charge transfer resistance, respectively.

### Simulated calculation

Uniaxial tensile simulations were performed on the single CLC fiber and single PVDF-HFP fiber using the Abaqus/Explicit dynamic analysis module. The single CLC fiber was modeled as a composite structure consisting of a single PVDF-HFP fiber and SiO_2_ particles, where multiple SiO_2_ particles were tied to the PVDF-HFP fiber via tie constraints. The fiber structure is simplified as a cylinder with a diameter of 0.005 mm and a length of 0.9 mm, while SiO_2_ is modeled as a solid sphere with a diameter of 0.003 mm. During tensile testing, one end is fixed, and the other end is stretched by 0.15 mm. Both the CLC and PVDF-HFP fibers were discretized using four-node linear tetrahedral solid elements (C3D4). The CLC fiber model consists of 5661 elements and 1840 nodes, while the PVDF-HFP fiber model comprises 2973 elements and 983 nodes.

The calculation of binding energy was achieved through the Forcite code in Materials Studio software. On the basis of the Compass II force field, the geometry optimization of all molecules or ions was carried out using Forcite code, and then the single-point energy of the optimized molecular or ionic structure was obtained by energy calculation of Forcite code. The formula is $E_{a}=E_{\text{total}}-E_{1}-E_{2}$, where $E_{\text{total}}$ represents the total energy of the molecules and ions in the system, and $E_{1}$ and $E_{2}$ are the energies of the single-component molecules or ions.

The Forcite code was used to conduct MD simulations on two electrolyte systems (PVDF-HFP GPE and CLC GPE). The detailed composition of the simulation systems is listed in table S4. All simulations selected the Compass II force field. The MD simulations were performed using the canonical ensemble (NVT) for 300 ps with a time step of 1 fs, and the temperature was controlled by the Nose-Hoover thermostat. Last, the radial distribution functions and coordination numbers were analyzed on the basis of the simulation results.

## Supplementary Materials

### The PDF file includes:

Figs. S1 to S43 Tables S1 to S4 Legends for movies S1 to S4 References
