# Supplementary material for: A caddisfly larva case–mimicked gel polymer electrolyte with high toughness and enhanced ion transport for safe lithium metal batteries
Source: Sci Adv. 2026 Jul 8;12(28):eaed3988. doi: 10.1126/sciadv.aed3988 (PMC13344280; doi:10.1126/sciadv.aed3988)
Supplement: Supplementary file 1 — Figs. S1 to S43 Tables S1 to S4 Legends for movies S1 to S4 References [file sciadv.aed3988_sm.pdf]

Science Advances
AAAS

# Supplementary Materials for

## A caddisfly larva case–mimicked gel polymer electrolyte with high toughness and enhanced ion transport for safe lithium metal batteries

Quan Liu *et al.*

Corresponding author: Bing Liu, bingliu@ustc.edu.cn; Huaxia Deng, hxdeng@ustc.edu.cn; Xinglong Gong, gongxl@ustc.edu.cn

*Sci. Adv.* **12**, eaed3988 (2026)
DOI: 10.1126/sciadv.aed3988

**The PDF file includes:**

Figs. S1 to S43
Tables S1 to S4
Legends for movies S1 to S4
References

**Other Supplementary Material for this manuscript includes the following:**

Movies S1 to S4

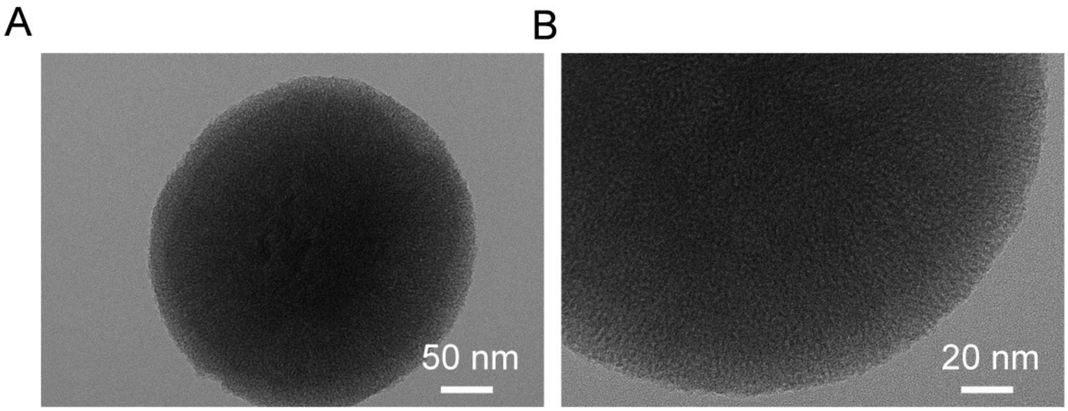


**Fig. S1. TEM analysis.** (**A**) TEM image of M-$SiO_2$. (**B**) The mesoporous structure of M-$SiO_2$.

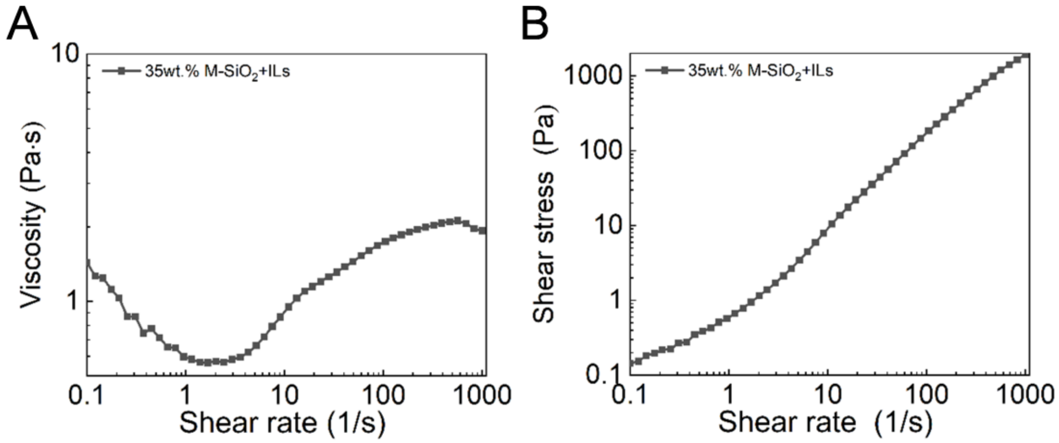


**Fig. S2. Rheological testing.** (**A**) The viscosity-shear rate curve and (**B**) the shear stress-shear rate curve of shear thickening behavior of STF.

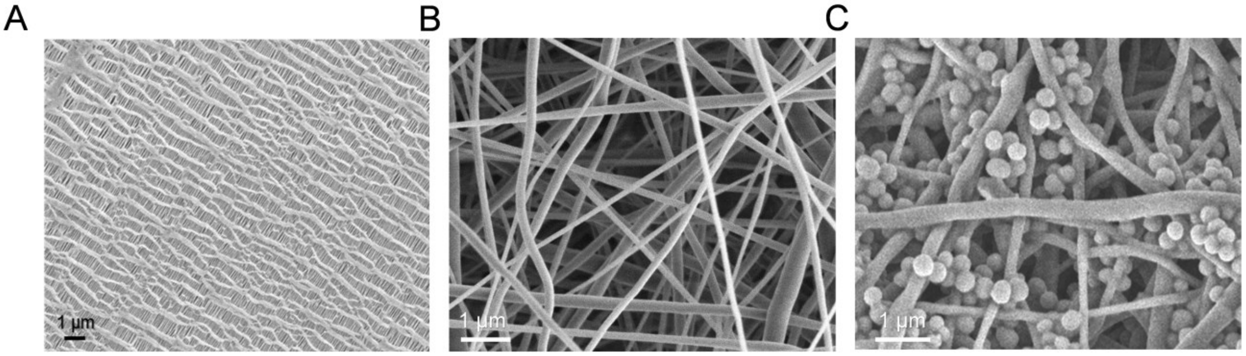


**Fig. S3. SEM analysis.** SEM image of (**A**) PP, (**B**) PVDF-HFP, and (**C**) CLC fiber membranes.

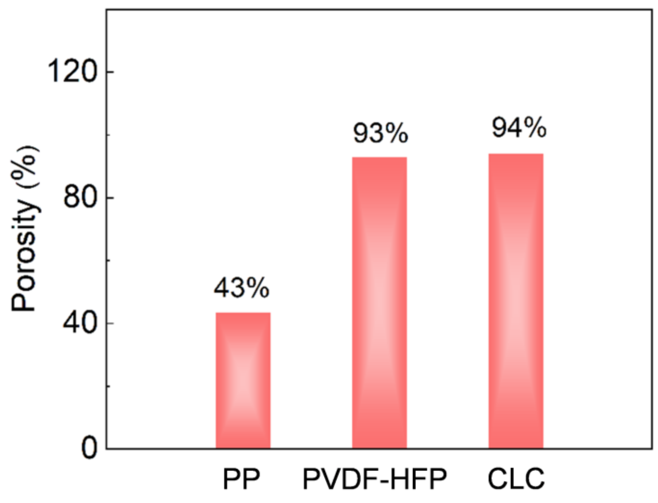


**Fig. S4.** Porosity of PP, PVDF-HFP, and CLC fiber membranes.

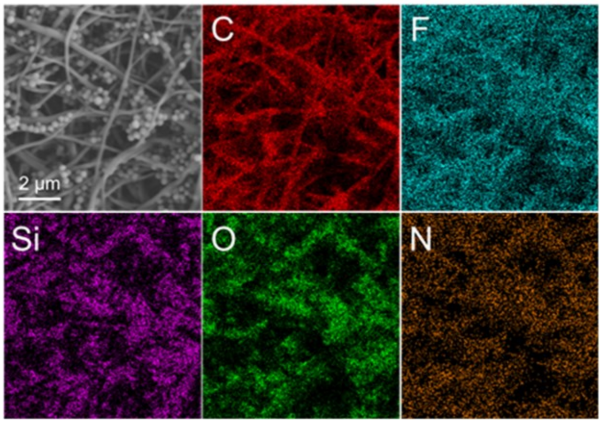


**Fig. S5.** EDS results of CLC.

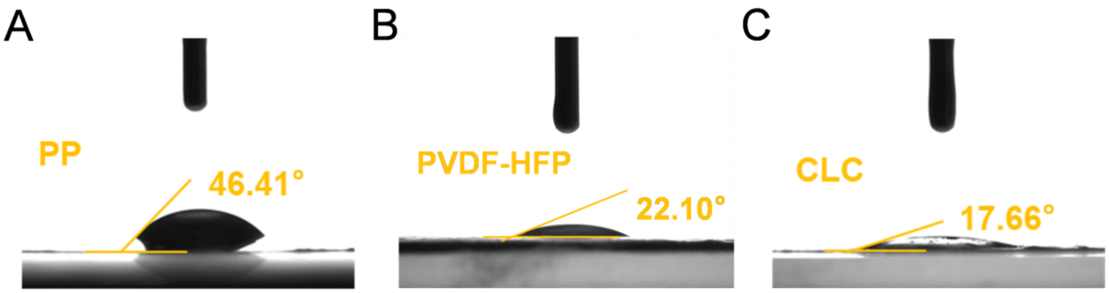


**Fig. S6. Contact angle test.** The results of the electrolyte contact angle of (**A**) PP, (**B**) PVDF-HFP, and (**C**) CLC fiber membranes.

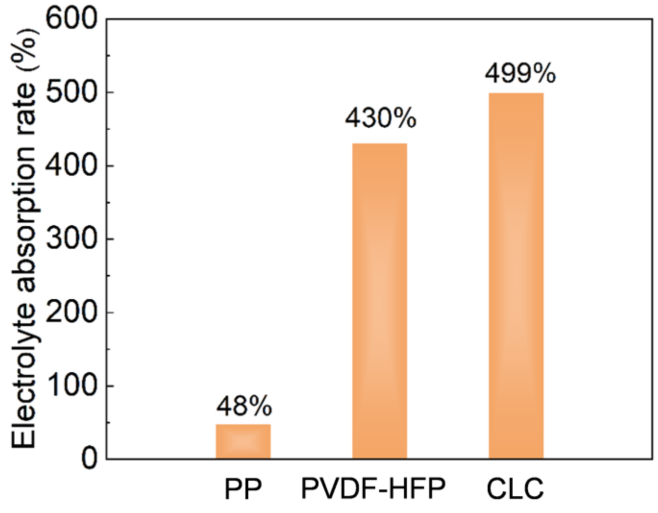


**Fig. S7.** Electrolyte absorption rate of PP, PVDF-HFP, and CLC fiber membranes.

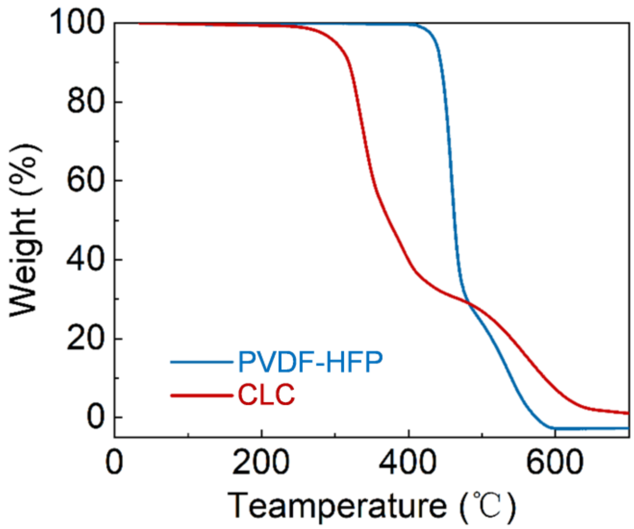


**Fig. S8.** TG results of PVDF-HFP and CLC fiber membranes.

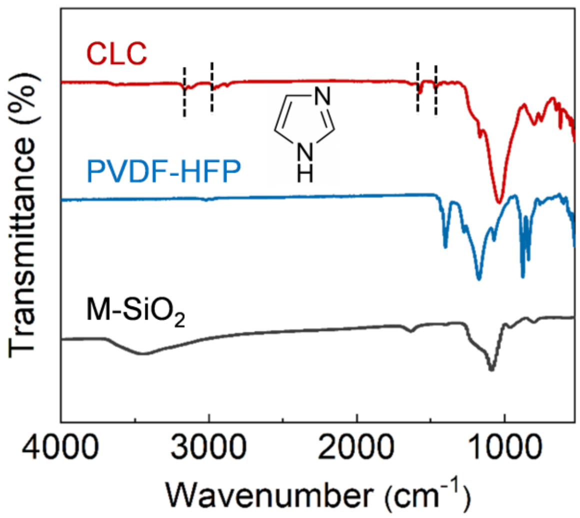


**Fig. S9.** FTIR spectra of CLC, PVDF-HFP, and M-$SiO_2$.

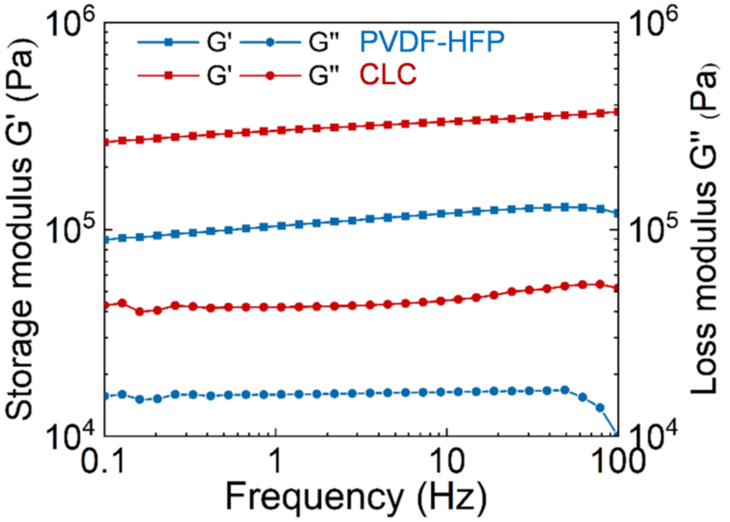


**Fig. S10.** Frequency-sweeping rheological curves of CLC and PVDF-HFP.

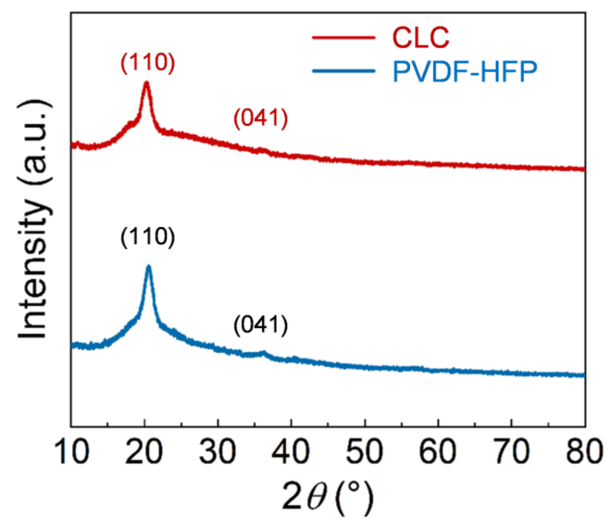


**Fig. S11.** XRD spectra of CLC and PVDF-HFP.

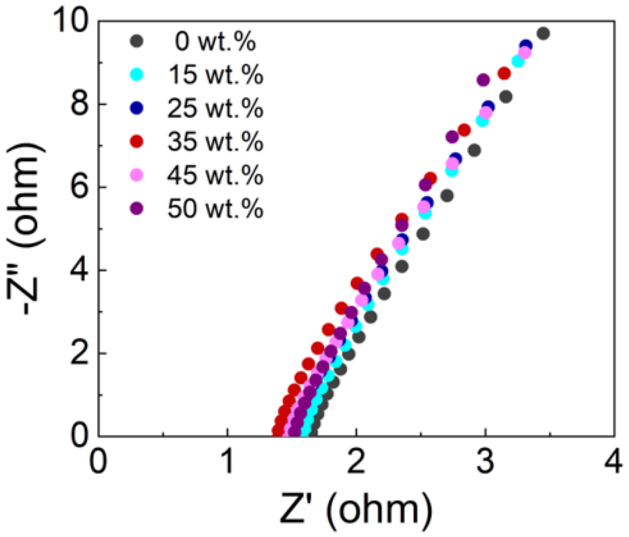


**Fig. S12.** EIS Nyquist plots of CLC GPE with different ratio of PVDF-HFP and STF at 25℃.

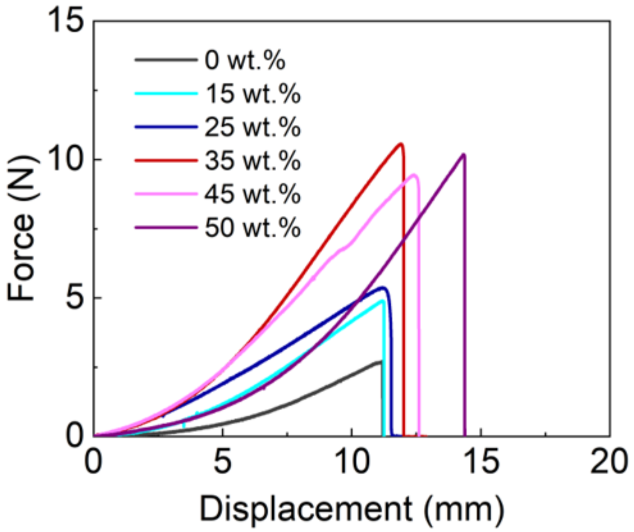


**Fig. S13.** Puncture force-displacement curves of CLC GPE with different ratio of PVDF-HFP and STF.

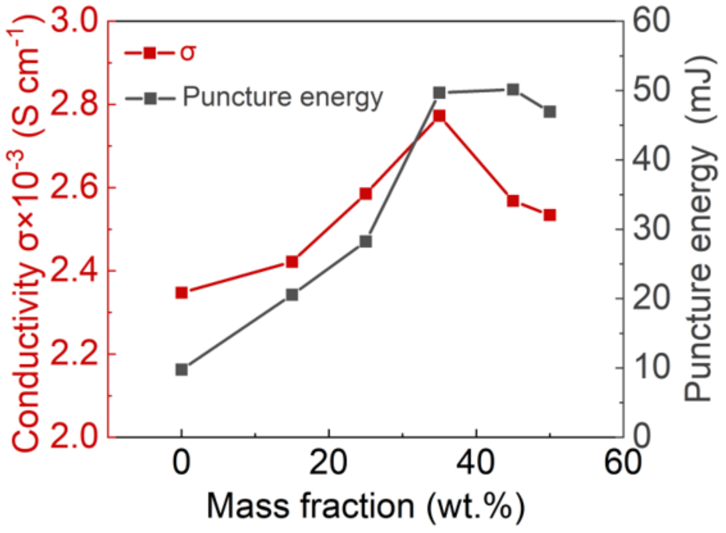


**Fig. S14.** The relationship curves of the ionic conductivity and puncture energy of CLC GPE with the composite ratio of STF.

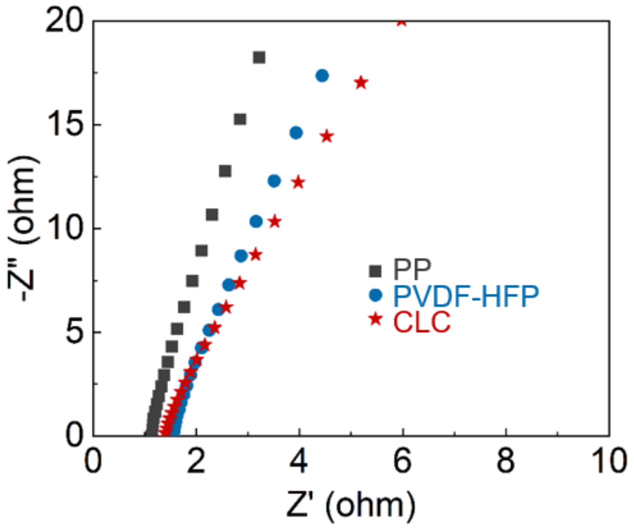


**Fig. S15.** EIS Nyquist plots of PP, PVDF-HFP, and CLC GPEs at 25℃.

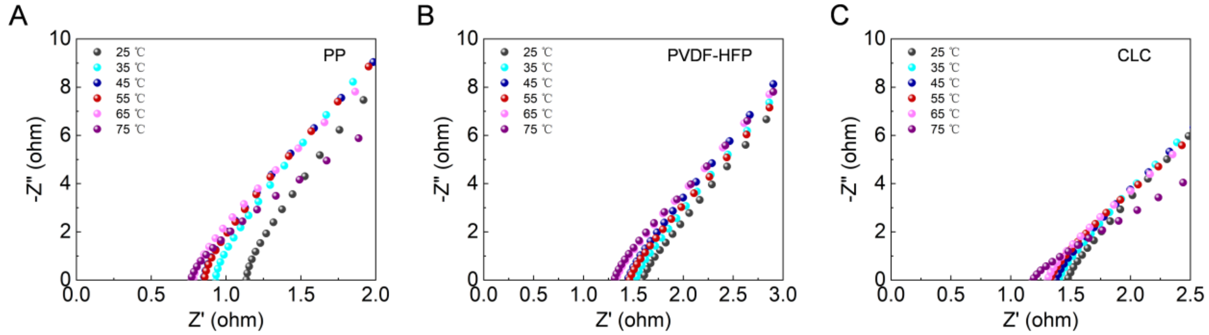


**Fig. S16. EIS analysis.** EIS Nyquist plots of (**A**) PP, (**B**) PVDF-HFP, and (**C**) CLC GPEs at different temperatures.

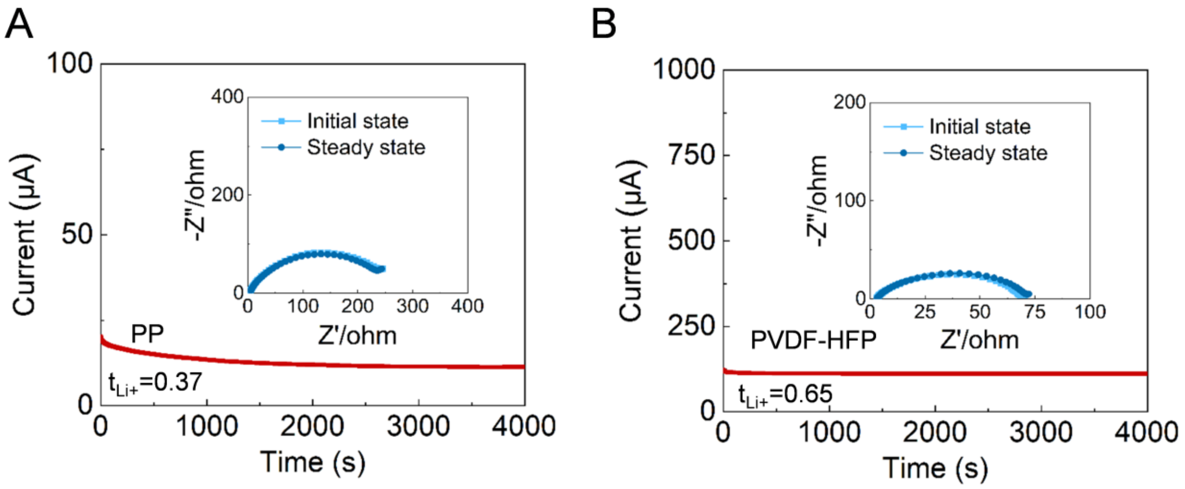


**Fig. S17. Tests of the Li$^+$ transference numbers.** Current-time plots of (**A**) PP and (**B**) PVDF-HFP GPE in Li||Li cells under a constant potential of 10 mV. Insert: Nyquist plots before and after polarization.

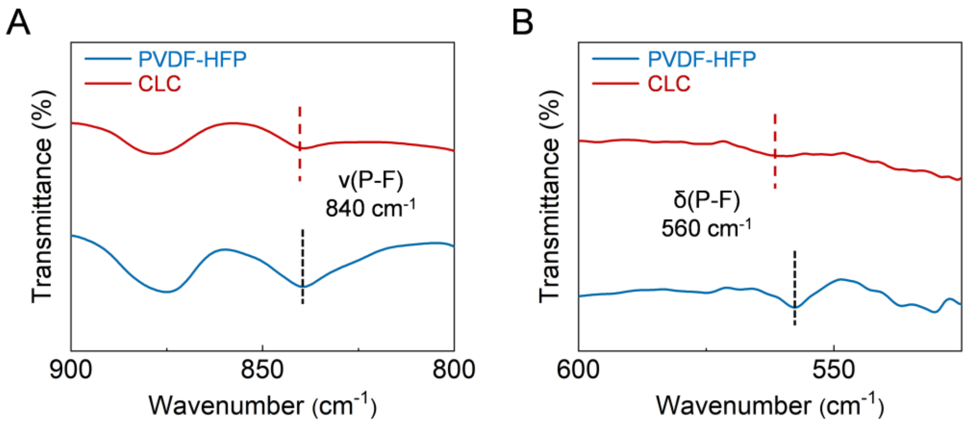


**Fig. S18. The results of ATR-FTIR tests of PVDF-HFP and CLC GPEs.** (**A**) The stretching vibration peak of $PF_6^-$ at 840 cm$^{-1}$. (**B**) The bending vibration peak at 560 cm$^{-1}$.

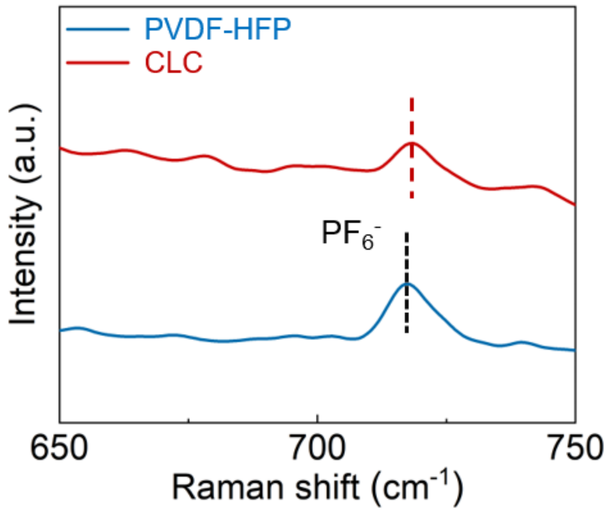


**Fig. S19.** The Raman spectra of PVDF-HFP and CLC GPEs.

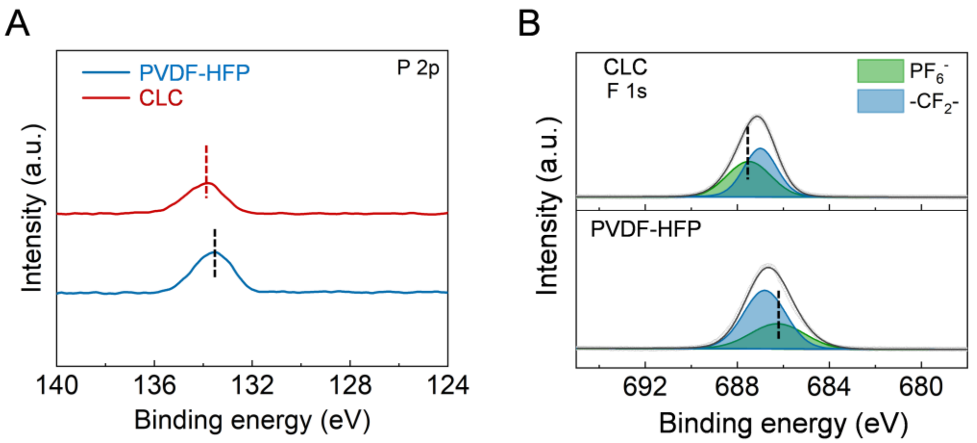


**Fig. S20. The results of XPS of PVDF-HFP and CLC GPEs.** The binding energy peaks in the (**A**) P 2p and (**B**) F 1s.

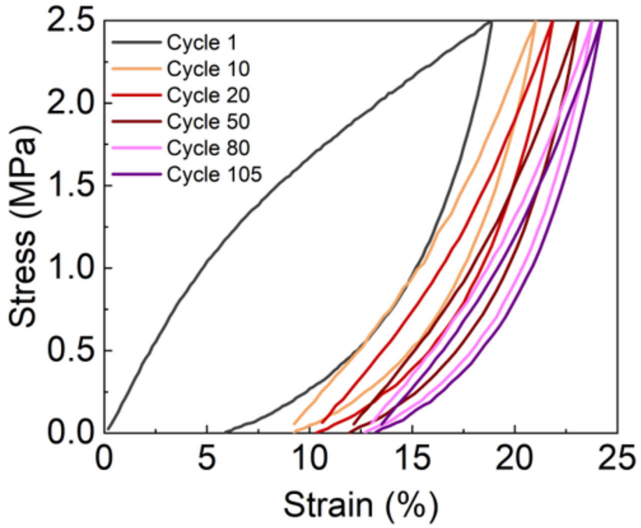


**Fig. S21.** The cyclic tensile curve of CLC under a maximum stress of 2.5 MPa.

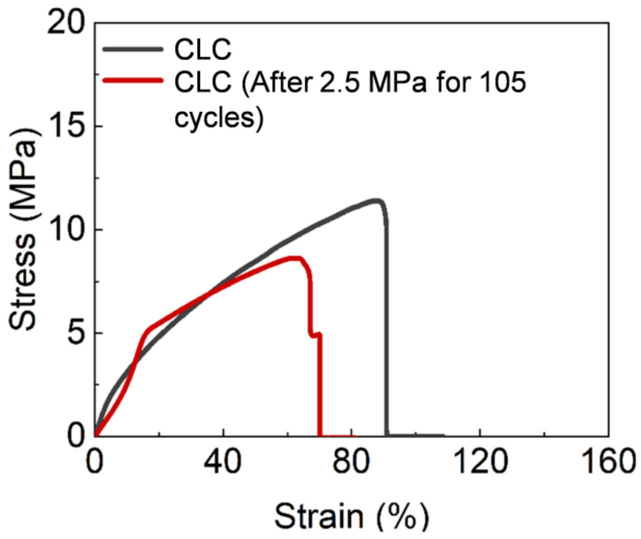


**Fig. S22.** Stress-strain curves of CLC fiber membranes before and after cyclic stretching.

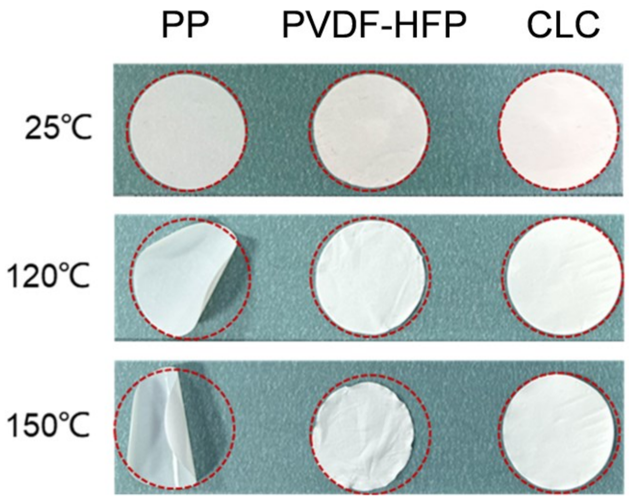


**Fig. S23.** The results of the thermal stability tests of PP, PVDF-HFP, and CLC membranes.

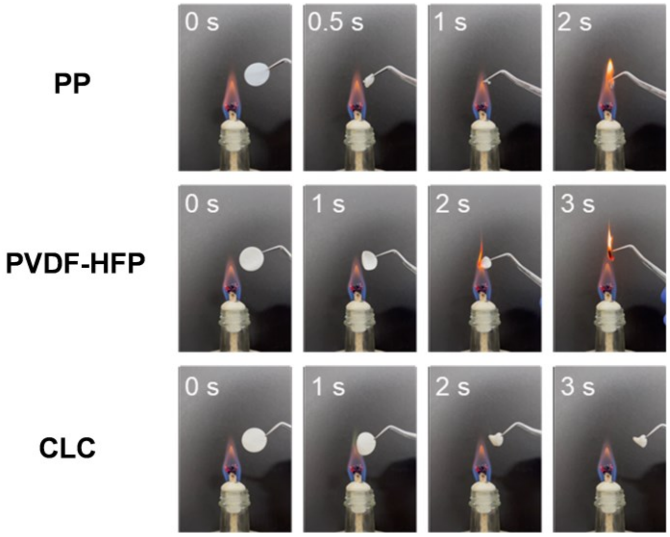


**Fig. S24.** Flame-retardant properties of PP, PVDF-HFP, and CLC.

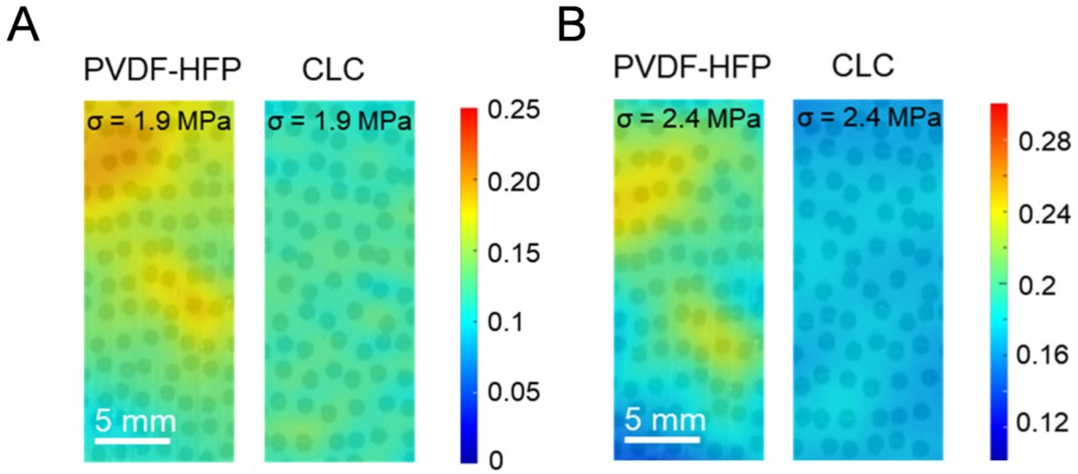


**Fig. S25. DIC analysis.** Strain field $\varepsilon_{yy}$ of PVDF-HFP and CLC at (**A**) σ=1.9 MPa and (**B**) σ=2.4 MPa.

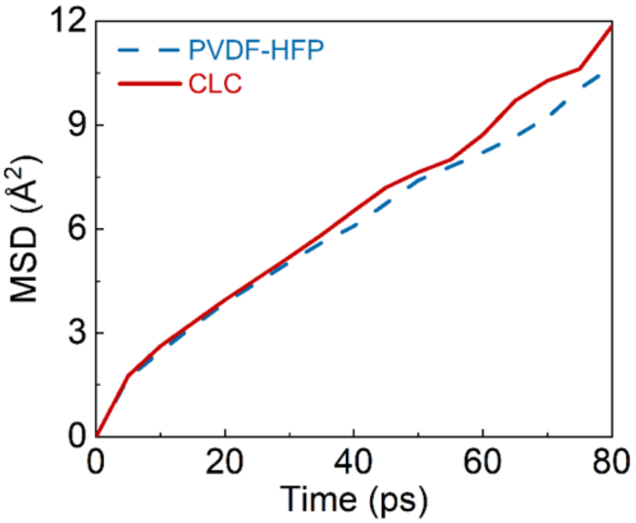


**Fig. S26.** $Li^+$ mean squared displacement in the CLC and PVDF-HFP GPEs simulation systems.

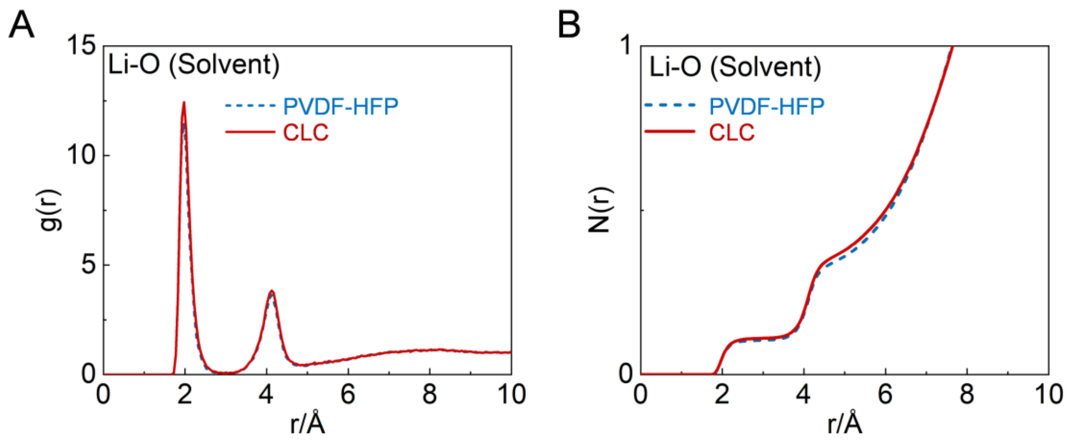


**Fig. S27. MD simulation results.** (**A**) Corresponding RDF plots and (**B**) coordination number of Li-O (Solvent)for two different electrolyte systems collected from molecular dynamics simulations.

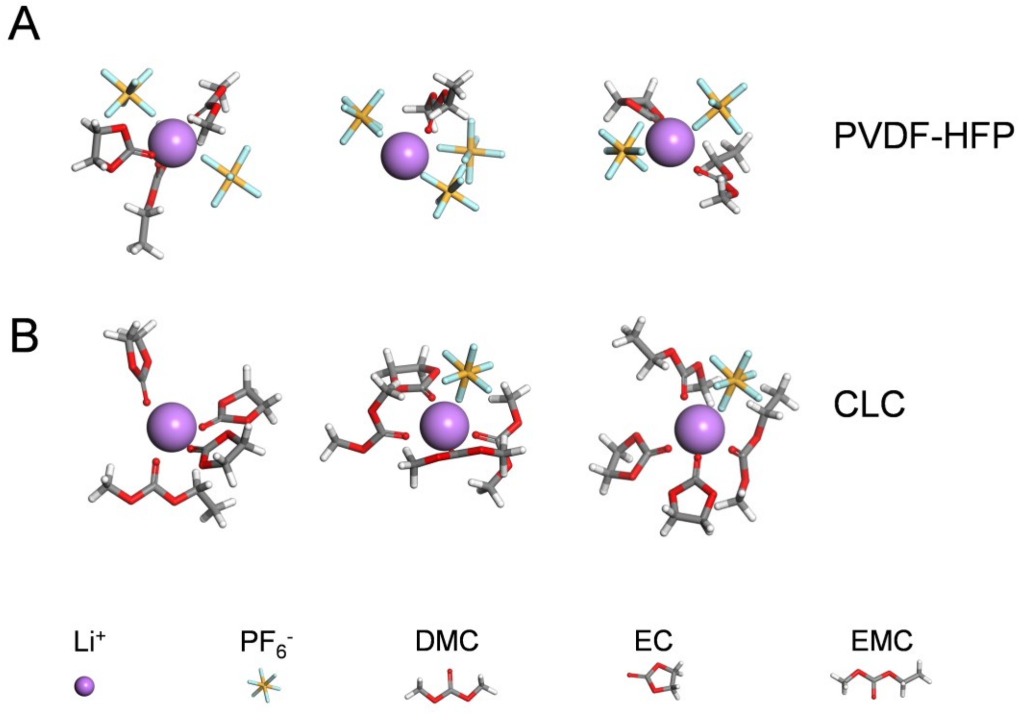


**Fig. S28. Coordination environment.** The coordination environment of $Li^+$ for (**A**) PVDF-HFP and (**B**) CLC GPEs.

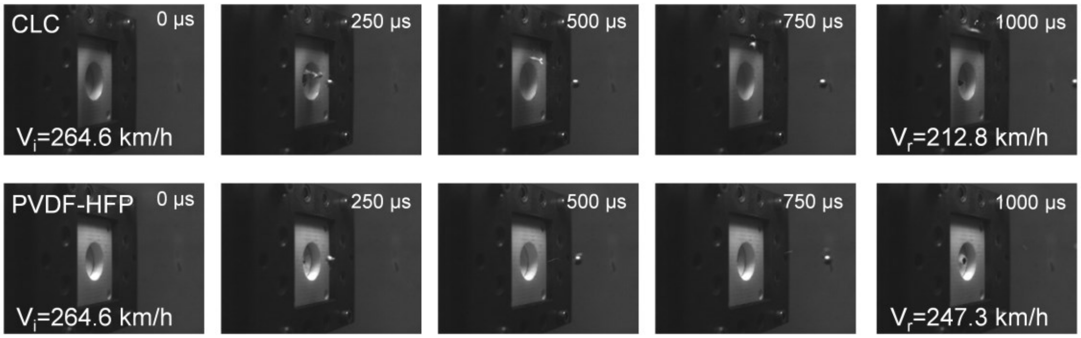


**Fig. S29.** The snapshots of bullet impact process of 264.6 km/h on PVDF-HFP and CLC membranes .

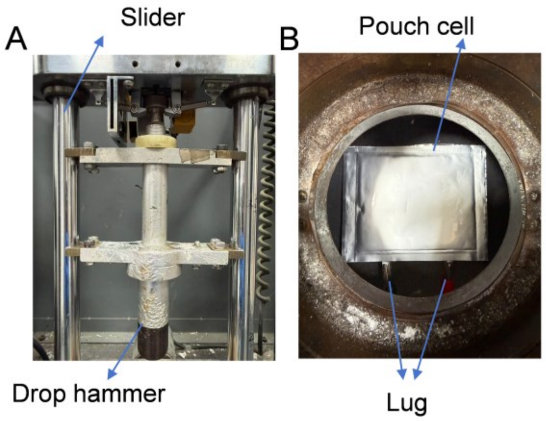


**Fig. S30. The drop hammer collision experiments.** (**A**) The devices of the drop hammer collision experiments. (**B**) The physical picture of the cell sample on the test bench.

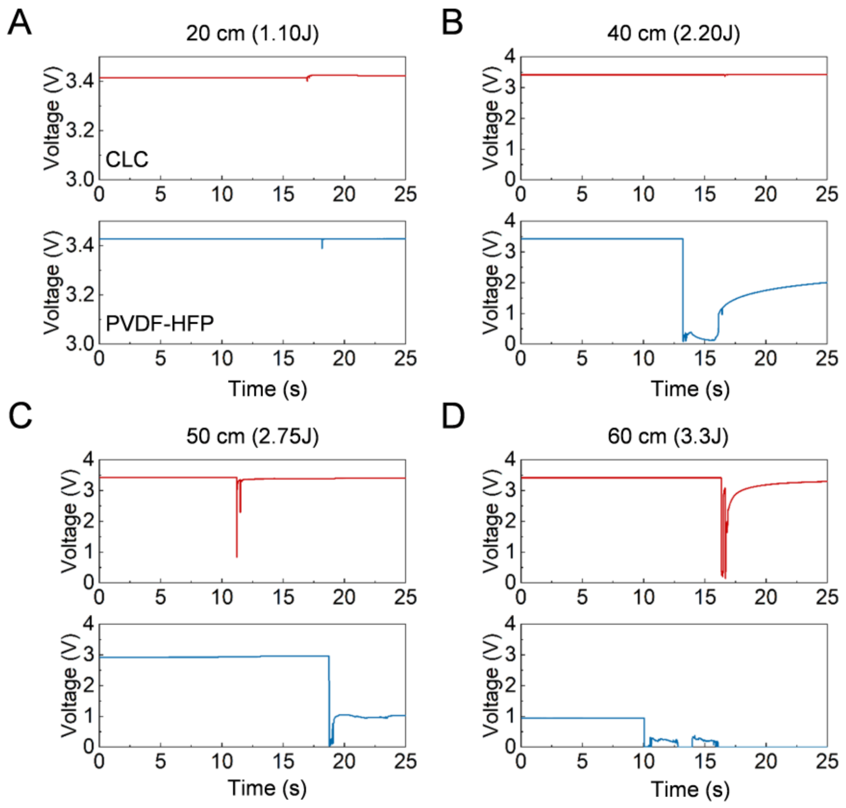


**Fig. S31. The results of the drop hammer collision experiments.** Voltage-time curves of two electrolyte pouch cells tested by drop hammer collision at different heights: (**A**) 20 cm, (**B**) 40 cm, (**C**) 50 cm, and (**D**) 60 cm.

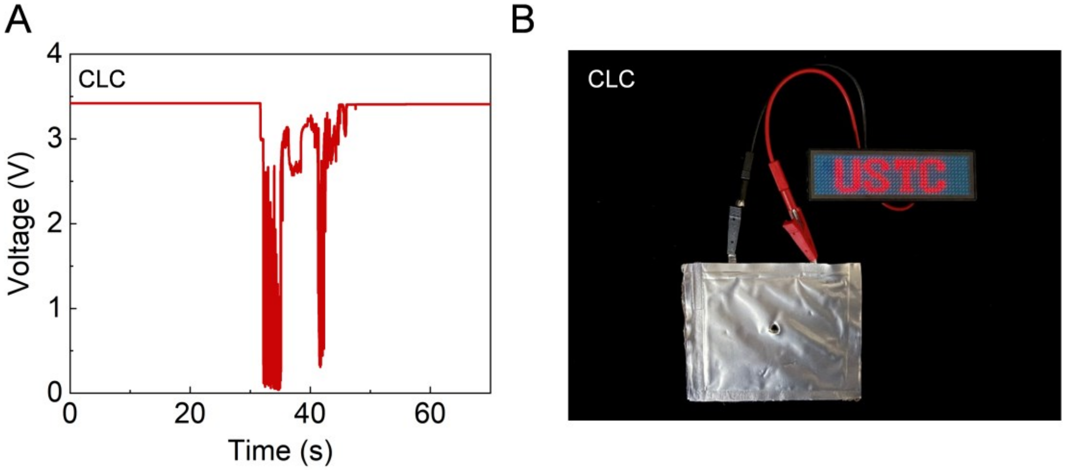


**Fig. S32. The nail penetration experiment.** (**A**) Voltage-time curve of the CLC GPE pouch cell tested by the nail penetration experiment. (**B**) The photo showing the resilience of the CLC GPE pouch cell to the nail penetration.

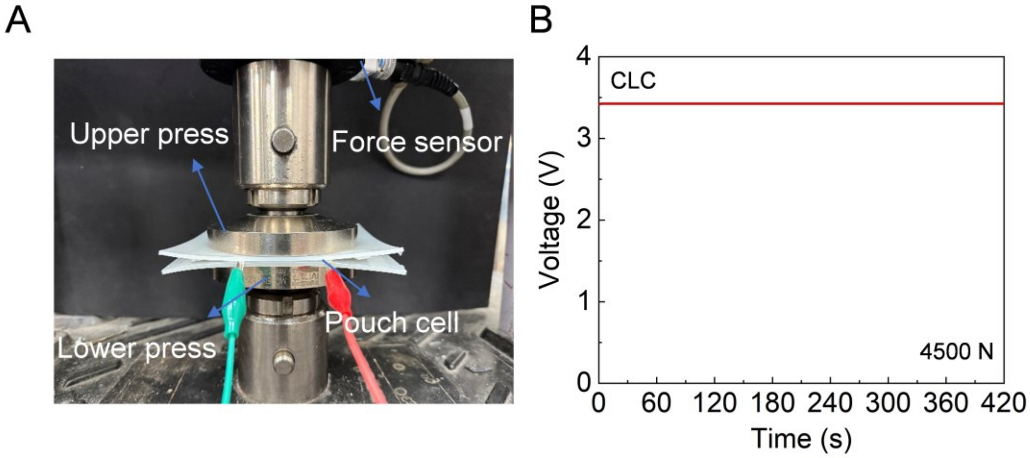


**Fig. S33. The compression experiment.** (**A)** The devices of the compression experiment. (**B**) The voltage-time curve of the pouch cell based on CLC GPE under an extrusion pressure of 4500 N.

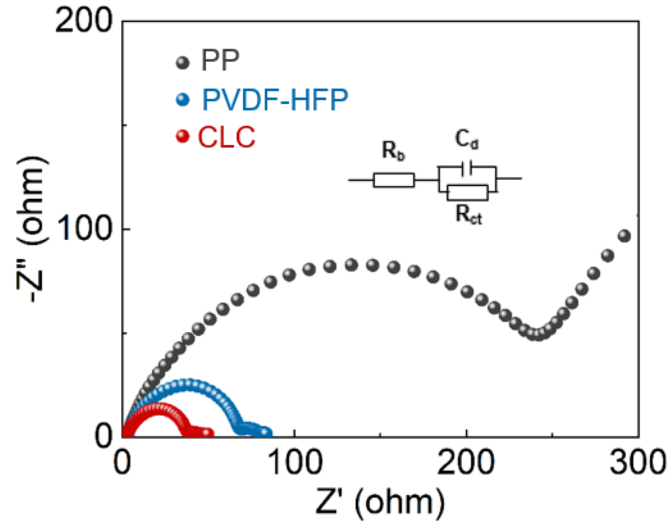


**Fig. S34.** Nyquist plots and equivalent circuit diagrams of Li||Li symmetric cells of PP, PVDF-HFP, and CLC GPEs.

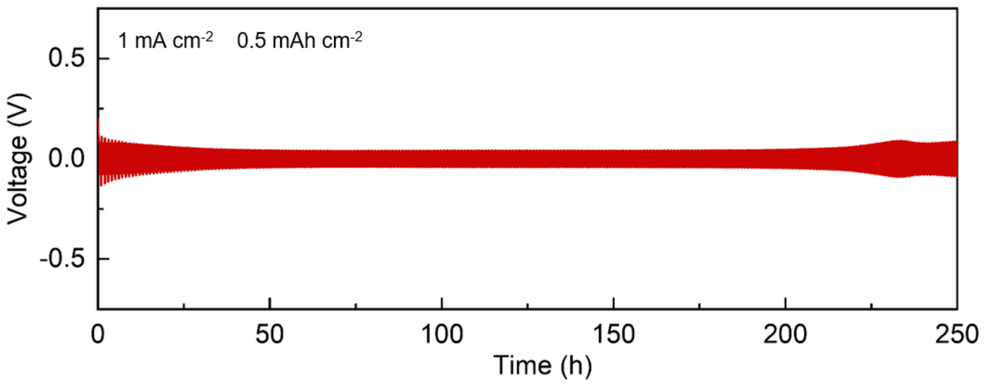


**Fig. S35.** Li plating/stripping cycling performance at 1 mA $cm^{-2}$ and 0.5 mAh $cm^{-2}$ using CLC GPE.

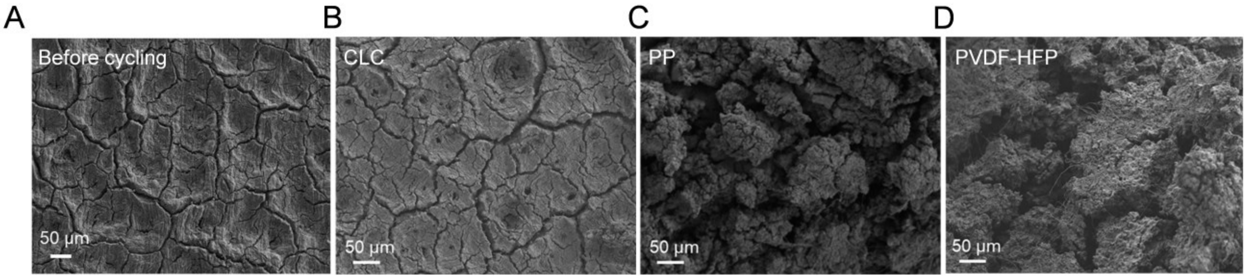


**Fig. S36. SEM analysis of the lithium anodes.** (**A**) SEM images of Li anodes before cycling. SEM images of Li anodes after cycling from (**B**) Li||CLC GPE||Li, (**C**) Li||PP||Li, and (**D**) Li||PVDF-HFP GPE||Li cells.

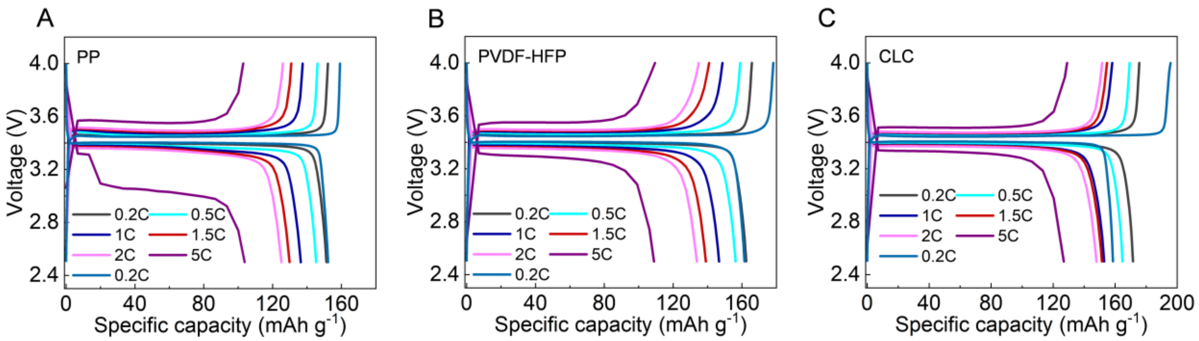


**Fig. S37. The electrochemical performance of full cells during rate test.** Charge–discharge curves of Li||LFP full cells during rate test of (**A**) PP, (**B**) PVDF-HFP, and (**C**) CLC GPEs.

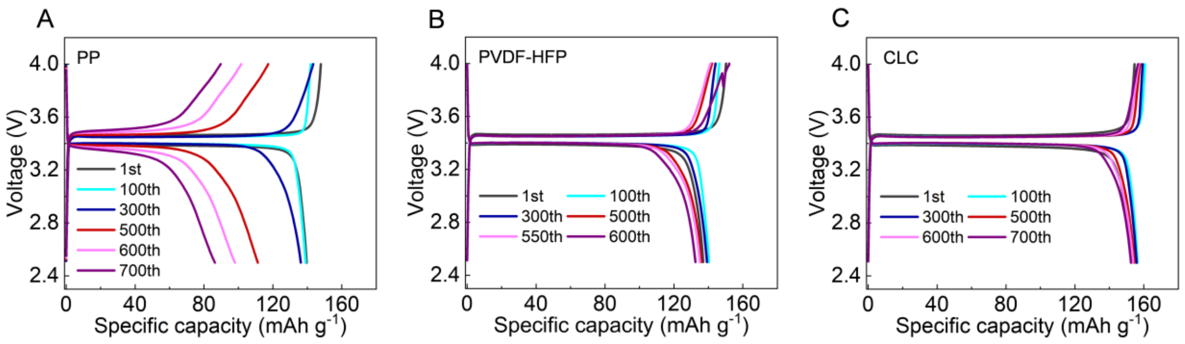


**Fig. S38. The cycling performance of full cells at 0.5C.** Charge–discharge curves of Li||LFP full cells during cycling test at 0.5C of (**A**) PP, (**B**) PVDF-HFP, and (**C**) CLC GPEs.

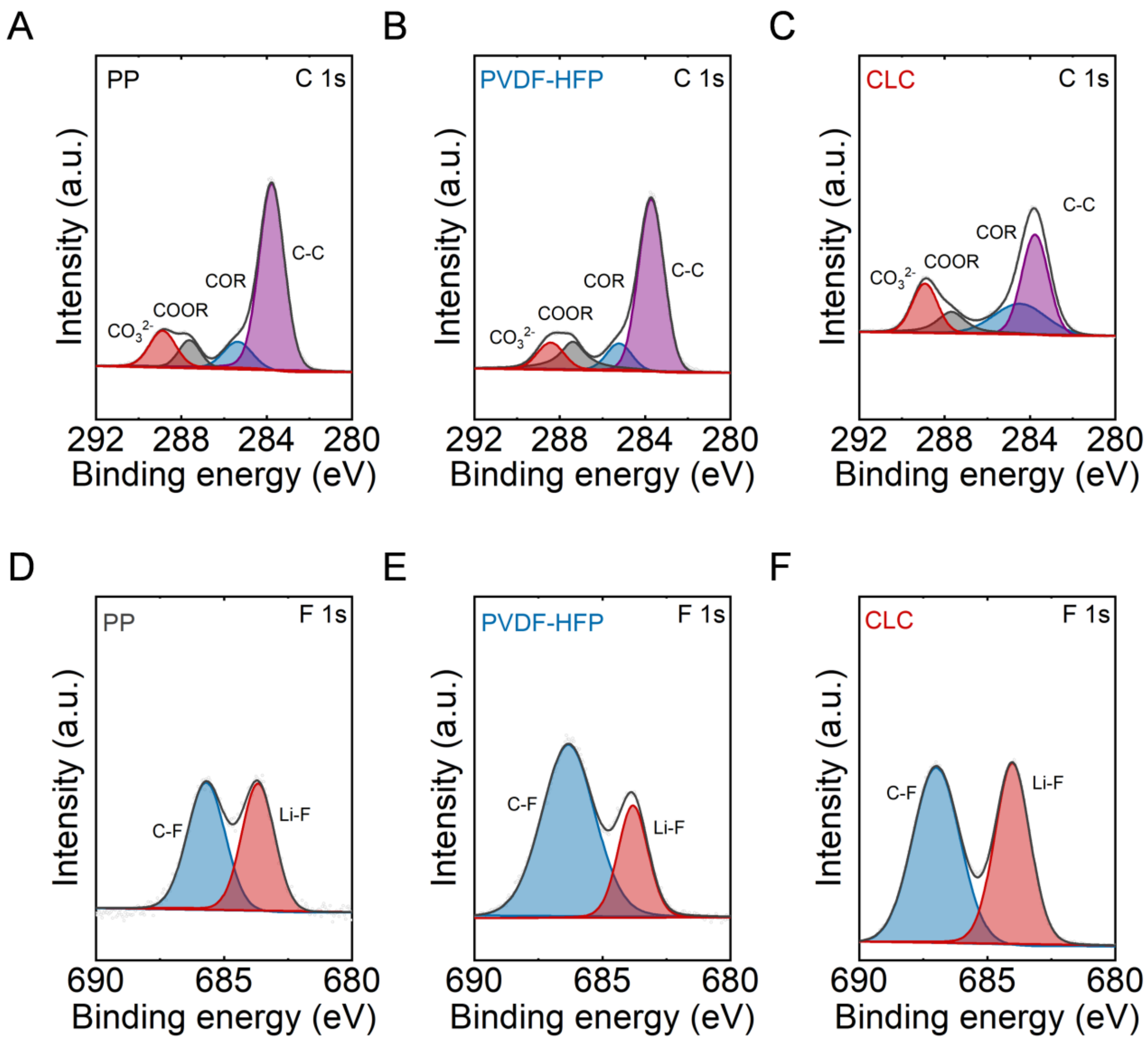


**Fig. S39. XPS spectra.** XPS spectra of C 1s for anodes retrieved from (**A**) PP, (**B**) PVDF-HFP, and (**C**) CLC GPEs before 50 cycles. XPS spectra of F 1s for anodes retrieved from (**D**) PP, (**E**) PVDF-HFP, and (**F**) CLC GPEs before 50 cycles.

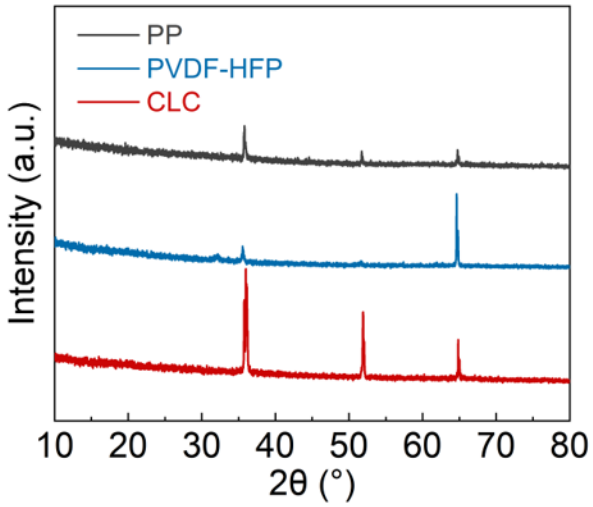

**Fig. S40.** XRD spectra for Li anodes retrieved from PP, PVDF-HFP, and CLC GPEs after 50 cycles.

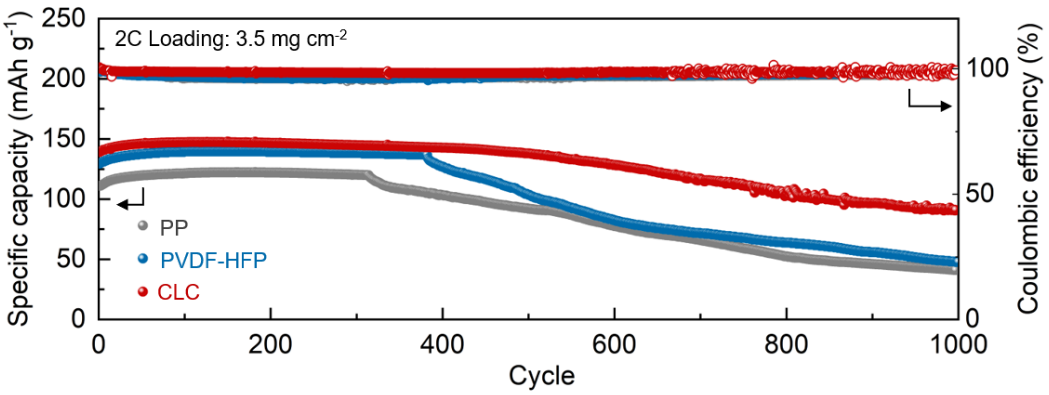


**Fig. S41.** Long-term cycling performance at 2C of Li||LFP cells of different GPEs.

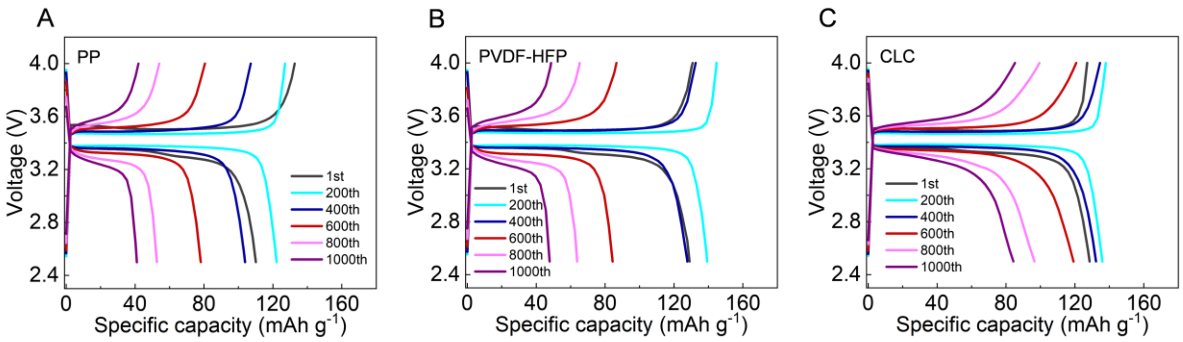


**Fig. S42. The cycling performance of full cells at 2C.** Charge–discharge curves of Li||LFP full cells during cycling test at 2C of (**A**) PP, (**B**) PVDF-HFP, and (**C**) CLC GPEs.

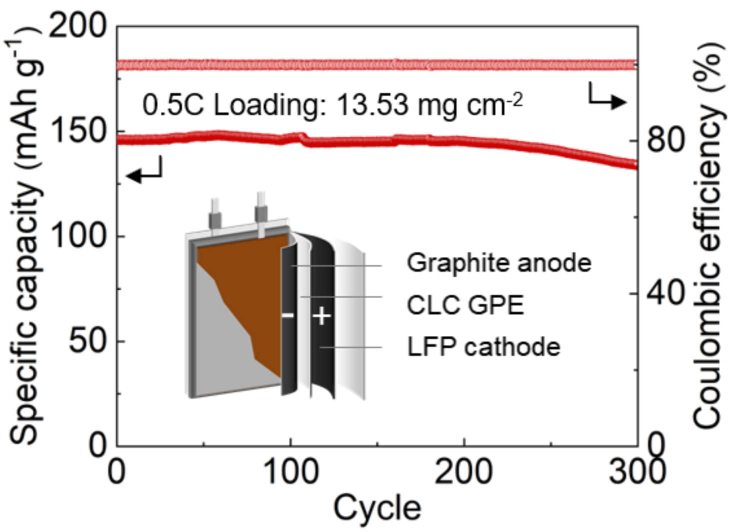


**Fig. S43.** Cycling performance for the CLC GPE pouch cell tested at 0.5C.

**Table S1. Literature comparison.** Comparative of ionic conductivity versus toughness across various reported polymer electrolytes.

| Samples | Toughness (MJ $m^{-3}$) | σ (mS $cm^{-1}$) | References |
|---|---|---|---|
| **CLC** | **7.29** | **2.80** | **This work** |
| PU-TFMSI-2 | 13.6 | 0.13 | Refer. 48 |
| eEPE | 3.4 | 0.35 | Refer. 49 |
| PUU-4 | 11.9 | 0.294 | Refer. 50 |
| PTL-1 | 0.585 | 0.11 | Refer. 51 |
| ePPO | 0.32 | 0.25 | Refer. 52 |
| PEO | 0.0463 | 0.02 | Refer. 52 |
| PEO 20 wt.% LAGP | 0.16 | 0.0212 | Refer. 52 |
| PEO 1 wt.% $SiO_2$ | 0.153 | 0.0054 | Refer. 53 |
| Aramid fiber in PEO | 7.69 | 0.005 | Refer. 54 |
| PU-PUDF | 10.55 | 0.512 | Refer. 55 |
| Crossing-MWPEGDA | 0.117 | 1.1 | Refer. 56 |
| Celgard 2325 | 1.8 | 0.62 | Refer. 57 |

**Table S2. Literature comparison.** Long-cycling performance with different GPEs compared with recent reports.

| Components of GPE | Cathode\|\| Anode | Rate | Cycle | Capacity retention (%) | Mass loading (mg $cm^{-2}$) | References |
|---|---|---|---|---|---|---|
| **CLC GPE** | **LFP\|\|Li** | **0.5C** | **700** | **97.6** | **3.5** | **This work** |
| PFP CA | LFP\|\|Li | 1C | 600 | 93 | / | Refer. 58 |
| AP-GPEs/LLZO-w | LFP\|\|Li | 1C | 600 | 87.3 | 2.0 | Refer. 59 |
| MB-GPE | LFP\|\|Li | 1C | 600 | 80.3 | 2.5 | Refer. 60 |
| GPE-PL | LFP\|\|Li | 0.5C | 500 | 71.6 | / | Refer. 61 |
| PLF@GPE | LFP\|\|Li | 0.5C | 500 | 91.4 | 3.9 | Refer. 62 |
| PDOL/$LiNO_3$ | LFP\|\|Li | 0.5C | 450 | 80.3 | 3.0 | Refer. 63 |
| TFGPE | LFP\|\|Li | 0.5C | 300 | 89.4 | 2.5 | Refer. 64 |
| PA1D1-GPE | LFP\|\|Li | 0.5C | 250 | 82.6 | 1.6 | Refer. 65 |
| TPP@PAN/PVDF-HFP GPE | LFP\|\|Li | 0.5C | 200 | 91.2 | 11 | Refer. 66 |
| PDOL@PDA/PVDF-HFP | LFP\|\|Li | 1C | 200 | 87.13 | 2.2 | Refer. 67 |
| BAEDA-PETEA-LE-PAN | LFP\|\|Li | 1C | 140 | 94 | 2.0 | Refer. 68 |
| PBz-PO-GPE 2000 | LFP\|\|Li | 0.5C | 100 | 90.2 | 2~4 | Refer. 69 |

**Table S3. The final composition of the CLC GPE.** The mass fractions of each component in the CLC GPE.

| Content | Mass fraction (wt.%) |
|---|---|
| PVDF-HFP fiber | 10.8 |
| M-$SiO_2$ | 2.0 |
| Ionic liquid | 3.9 |
| Liquid electrolyte | 83.3 |

**Table S4. Parameters related to MD simulations.** Detailed simulation of PVDF-HFP GPE and CLC GPE system compositions.

| | PVDF-HFP GPE | CLC GPE |
|---|---|---|
| EC | 479 | 479 |
| DMC | 379 | 379 |
| EMC | 309 | 309 |
| $LiPF_6$ | 100 | 100 |
| PVDF-HFP | 10 (for PVDF-HFP 5-mer) | 10 (for PVDF-HFP 5-mer) |
| $BMIMBF_4$ | / | 5 |
| $SiO_2$ | / | Filled in in cluster form |

**Movie S1.** The impact process of a bullet impacting the PVDF-HFP membrane at an incident velocity of 150.1 km/h.

**Movie S2.** The impact process of a bullet impacting the CLC membrane at an incident velocity of 150.1 km/h.

**Movie S3.** The impact process of a bullet impacting the PVDF-HFP membrane at an incident velocity of 264.6 km/h.

**Movie S4.** The impact process of a bullet impacting the CLC membrane at an incident velocity of 264.6 km/h.

## REFERENCES


1. Q. Kang, Z. Zhuang, Y. Liu, Z. Liu, Y. Li, B. Sun, F. Pei, H. Zhu, H. Li, P. Li, Y. Lin, K. Shi, Y. Zhu, J. Chen, C. Shi, Y. Zhao, P. Jiang, Y. Xia, D. Wang, X. Huang, Engineering the structural uniformity of gel polymer electrolytes via pattern-guided alignment for durable, safe solid-state lithium metal batteries. *Adv. Mater.* **35**, e2303460 (2023).

2. W. Hong, A. Wang, L. Li, T. Qiu, J. Li, Y. Jiang, G. Zou, H. Peng, H. Hou, X. Ji, Bi dots confined by functional carbon as high-performance anode for lithium ion batteries. *Adv. Funct. Mater.* **31**, 2000756 (2021).

3. M. J. Lee, J. Han, K. Lee, Y. J. Lee, B. G. Kim, K. N. Jung, B. J. Kim, S. W. Lee, Elastomeric electrolytes for high-energy solid-state lithium batteries. *Nature* **601**, 217–222 (2022).

4. X. Shen, H. Liu, X. Cheng, C. Yan, J. Huang, Beyond lithium ion batteries: Higher energy density battery systems based on lithium metal anodes. *Energy Storage Mater.* **12**, 161–175 (2018).

5. X. Hu, K. Liu, S. Zhang, G. Shao, S. R. P. Silva, P. Zhang, A functional gel polymer electrolyte based on PVDF-HFP/gelatin toward dendrite-free lithium metal batteries. *Nano Res.* **17**, 2824–2835 (2024).

6. X. Pei, Y. Li, T. Ou, X. Liang, Y. Yang, E. Jia, Y. Tan, S. Guo, Li-N interaction induced deep eutectic gel polymer electrolyte for high performance lithium-metal batteries. *Angew. Chem. Int. Ed. Engl.* **61**, e202205075 (2022).

7. L. Han, C. Liao, Y. Liu, H. Yu, S. Zhang, Y. Zhu, Z. Li, X. Li, Y. Kan, Y. Hu, Non-flammable sandwich-structured TPU gel polymer electrolyte without flame retardant addition for high performance lithium ion batteries. *Energy Storage Mater.* **52**, 562–572 (2022).

8. S. Yang, X. He, T. Hu, Y. He, S. Lv, Z. Ji, Z. Zhu, X. Fu, W. Yang, Y. Wang, A supertough, nonflammable, biomimetic gel with neuron-like nanoskeleton for puncture-tolerant safe lithium metal batteries. *Adv. Funct. Mater.* **33**, 2304727 (2023).

9. J. Song, K. Liao, J. Si, C. Zhao, J. Wang, M. Zhou, H. Liang, J. Gong, Y. Cheng, J. Gao, Y. Xia, Phosphonate-functionalized ionic liquid gel polymer electrolyte with high safety for dendrite-free lithium metal batteries. *ACS Appl. Mater. Interfaces* **15**, 2901–2910 (2023).

10. H. Hong, Z. Wei, Y. Wang, X. Yang, X. Guo, Q. Nian, X. Li, Q. Li, S. Wang, S. Li, D. Zhang, Q. Xiong, Z. Huang, C. Zhi, A solid-state battery capable of 180 C superfast charging and 100% energy retention at −30 °C. *Proc. Natl. Acad. Sci. U.S.A.* **122**, e2511121122 (2025).

11. S. Han, P. Wen, H. Wang, Y. Zhou, Y. Gu, L. Zhang, Y. Shao-Horn, X. Lin, M. Chen, Sequencing polymers to enable solid-state lithium batteries. *Nat. Mater.* **22**, 1515–1522 (2023).

12. C. Zhao, X. Zhang, X. Cheng, R. Zhang, R. Xu, P. Chen, H. Peng, J. Huang, Q. Zhang, An anion-immobilized composite electrolyte for dendrite-free lithium metal anodes. *Proc. Natl. Acad. Sci. U.S.A.* **114**, 11069–11074 (2017).

13. K. Hashimoto, T. Shiwaku, H. Aoki, H. Yokoyama, K. Mayumi, K. Ito, Strain-induced crystallization and phase separation used for fabricating a tough and stiff slide-ring solid polymer electrolyte. *Sci. Adv.* **9**, eadi8505 (2023).

14. X. Deng, J. Chen, X. Jia, X. Da, Y. Zhao, Y. Gao, X. Kong, S. Ding, G. Gao, Highly tough slide-crosslinked gel polymer electrolyte for stable lithium metal batteries. *Angew. Chem. Int. Ed. Engl.* **63**, e202410818 (2024).

15. X. Mu, X. Li, C. Liao, H. Yu, Y. Jin, B. Yu, L. Han, L. Chen, Y. Kan, L. Song, Y. Hu, Phosphorus-fixed stable interfacial nonflammable gel polymer electrolyte for safe flexible lithium-ion batteries. *Adv. Funct. Mater.* **32**, 2203006 (2022).

16. R. Huang, R. Xu, J. Zhang, J. Wang, T. Zhou, M. Liu, X. Wang, PVDF-HFP-SN-based gel polymer electrolyte for high-performance lithium-ion batteries. *Nano Res.* **16**, 9480–9487 (2023).

17. J. Zhu, J. Zhang, R. Zhao, Y. Zhao, J. Liu, N. Xu, X. Wan, C. Li, Y. Ma, H. Zhang, Y. Chen, In situ 3D crosslinked gel polymer electrolyte for ultra-long cycling, high-voltage, and high-safety lithium metal batteries. *Energy Storage Mater.* **57**, 92–101 (2023).

18. B. Scrosati, J. Hassoun, Y. K. Sun, Lithium-ion batteries. A look into the future. *Energy Environ. Sci.* **4**, 3287–3295 (2011).

19. J. Zhang, J. Zhao, L. Yue, Q. Wang, J. Chai, Z. Liu, X. Zhou, H. Li, Y. Guo, G. Cui, L. Chen, Safety-reinforced poly (propylene carbonate)-based all-solid-state polymer electrolyte for ambient-temperature solid polymer lithium batteries. *Adv. Energy Mater.* **5**, 1501082 (2015).

20. L. Feng, Y. Xu, J. Wu, B. Lin, Semi-interpenetrating polymer network-based gel polymer electrolytes for Li-ion batteries applications. *J. Electroanal. Chem.* **978**, 118885 (2025).

21. Y. Zhang, X. Zhang, S. R. P. Silva, B. Ding, P. Zhang, G. Shao, Lithium-sulfur batteries meet electrospinning: Recent advances and the key parameters for high gravimetric and volume energy density. *Adv. Sci.* **9**, e2103879(2022).

22. H. Yang, Z. Liu, Y. Wang, N. Li, L. Yu, Multiscale structural gel polymer electrolytes with fast $Li^+$ transport for long-life Li metal batteries. *Adv. Funct. Mater.* **33**, 2209837 (2023).

23. S. Liu, W. Liu, D. Ba, Y. Zhao, Y. Ye, Y. Li, J. Liu, Filler-integrated composite polymer electrolyte for solid-state lithium batteries. *Adv. Mater.* **35**, e2110423 (2023).

24. K. Fu, Y. Gong, J. Dai, A. Gong, X. Han, Y. Yao, C. Wang, Y. Wang, Y. Chen, C. Yan, Y. Li, E. D. Wachsman, L. Hu, Flexible, solid-state, ion-conducting membrane with 3D garnet nanofiber networks for lithium batteries. *Proc. Natl. Acad. Sci. U.S.A.* **113**, 7094–7099 (2016).

25. M. Wang, Y. Wu, M. Qiu, X. Li, C. Li, R. Li, J. He, G. Lin, Q. Qian, Z. Wen, X. Li, Z. Wang, Q. Chen, Q. Chen, J. H. Lee, Y. Mai, Y. Chen, Research progress in electrospinning engineering for all-solid-state electrolytes of lithium metal batteries. *J. Energy Chem.* **61**, 253–268 (2021).

26. X. Li, Y. Deng, K. Li, Z. Yang, X. Hu, Y. Liu, Z. Zhang, Advancements in performance optimization of electrospun polyethylene oxide-Based solid-State electrolytes for lithium-ion batteries. *Polymers* **15**, 3727 (2023).

27. X. He, Y. Ni, W. Ma, Q. Zhang, Z. Hao, Y. Hou, H. Li, Z. Yan, K. Zhang, J. Chen, PVDF-HFP@Nafion-based quasisolid polymer electrolyte for high migration number in working rechargeable Na-$O_2$ batteries. *Proc. Natl. Acad. Sci. U.S.A.* **121**, e2320012121 (2024).

28. J. Sheng, Q. Zhang, C. Sun, J. Wang, X. Zhong, B. Chen, C. Li, R. Gao, Z. Han, G. Zhou, Crosslinked nanofiber-reinforced solid-state electrolytes with polysulfide fixation effect towards high safety flexible lithium-sulfur batteries. *Adv. Funct. Mater.* **32**, 2203272 (2022).

29. J. He, L. Yang, X. Ruan, Z. Liu, K. Liao, Q. Duan, Y. Zhan, Electrospun PVDF-based polymers for lithium-ion battery separators: A review. *Polymers* **16**, 2895 (2024).

30. Y. Li, Q. Li, Z. Tan, A review of electrospun nanofiber-based separators for rechargeable lithium-ion batteries. *J. Power Sources* **443**, 227262 (2019).

31. J. Zhu, C. Chen, Y. Lu, J. Zang, M. Jiang, D. Kim, X. Zhang, Highly porous polyacrylonitrile/graphene oxide membrane separator exhibiting excellent anti-self-discharge feature for high-performance lithium–sulfur batteries. *Carbon* **101**, 272–280 (2016).

32. C. Niu, G. Zhou, Q. Zhang, Z. Wei, Y. Kong, S. Zhang, X. Wang, L. Hou, Core-shell structured polymer electrolyte membranes for high absorption of liquid electrolytes and rapid transport of lithium ions: Design, preparation and characterization. *Chem. Eng. J.* **522**, 166961 (2025).

33. A. Mahmood, S. Li, Z. S. Ali, H. Tabassum, B. Zhu, Z. Liang, W. Meng, W. Aftab, W. Guo, H. Zhang, M. Yousaf, S. Gao, R. Zou, Y. Zhao, Ultrafast sodium/potassium-ion intercalation into hierarchically porous thin carbon shells. *Adv. Mater.* **31**, e1805430 (2019).

34. Q. Wu, M. Luo, J. Han, W. Peng, Y. Zhao, D. Chen, M. Peng, J. Liu, F. M. F. de Groot, Y. Tan, Identifying electrocatalytic sites of the nanoporous copper-ruthenium alloy for hydrogen evolution reaction in alkaline electrolyte. *ACS Energy Lett.* **5**, 192–199 (2020).

35. H. Kondo, K. Iimura, Thin film properties of thermally stable protic ionic liquids. *Bull. Chem. Soc. Jpn.* **94**, 2054–2059 (2021).

36. Y. Pei, Y. Zhang, J. Ma, M. Fan, S. Zhang, J. Wang, Ionic liquids for advanced materials. *Mater. Today Nano* **17**, 100159 (2022).

37. S. Zhang, Q. Zhang, Y. Zhang, Z. Chen, M. Watanabe, Y. Deng, Beyond solvents and electrolytes: Ionic liquids-based advanced functional materials. *Prog. Mater. Sci.* **77**, 80–124 (2016).

38. S. Roy, P. Thakur, N. A. Hoque, B. Bagchi, S. Das, Enhanced electroactive β-phase nucleation and dielectric properties of PVdF-HFP thin films influenced by montmorillonite and $Ni(OH)_2$ nanoparticle modified montmorillonite. *RSC Adv.* **6**, 21881–21894 (2016).

39. G. Zheng, T. Yan, Y. Hong, X. Zhang, J. Wu, Z. Liang, Z. Cui, L. Du, H. Song, A non-Newtonian fluid quasi-solid electrolyte designed for long life and high safety Li-$O_2$ batteries. *Nat. Commun.* **14**, 2268 (2023).

40. Y. Zhang, R. Rohan, W. Cai, G. Xu, Y. Sun, A. Lin, H. Cheng, Influence of chemical microstructure of single-ion polymeric electrolyte membranes on performance of lithium-ion batteries. *ACS Appl. Mater. Interfaces* **6**, 17534–17542 (2014).

41. Z. Lin, X. Guo, Z. Wang, B. Wang, S. He, L. A. O'Dell, J. Huang, H. Li, H. Yu, L. Chen, A wide-temperature superior ionic conductive polymer electrolyte for lithium metal battery. *Nano Energy* **73**, 104786 (2020).

42. J. Li, M. Jing, R. Li, L. Li, Z. Huang, H. Yang, M. Liu, S. Hussain, J. Xiang, X. Shen, $Al_2O_3$ fiber-reinforced polymer solid electrolyte films with excellent lithium-ion transport properties for high-voltage solid- state lithium batteries. *ACS Appl. Polym. Mater.* **4**, 7144–7151 (2022).

43. H. Yang, Z. Wang, X. Zhu, Z. Xu, J. Li, B. Luo, B. Guo, K. Wang, Enabling enhanced ion transport in PVDF-HFP electrolytes by engineering local dielectric microenvironments with dual-functional $SrTiO_3$/ LLZTO fillers. *Chem. Eng. J.* **525**, 170151 (2025).

44. D. Parikh, C. Jafta, B. Thapaliya, J. Sharma, H. Meyer, C. Silkowski, J. Li, $Al_2O_3/TiO_2$ coated separators: Roll-to-roll processing and implications for improved battery safety and performance. *J. Power Sources* **507**, 230259 (2021).

45. R. F. Recht, T. W. Ipson, Ballistic perforation dynamics. *J. Appl. Mech.* **30**, 384–390 (1963).

46. S. Zhang, B. Cheng, Y. Fang, D. Dang, X. Shen, Z. Li, M. Wu, Y. Hong, Q. Liu, Inhibition of lithium dendrites and dead lithium by an ionic liquid additive toward safe and stable lithium metal anodes. *Chin. Chem. Lett.* **33**, 3951–3954 (2022).

47. F. Wang, M. Liu, C. Liu, C. Huang, L. Zhang, A. Cui, Z. Hu, X. Du, Light control of droplets on photo-induced charged surfaces. *Natl. Sci. Rev.* **10**, nwac164 (2023).

48. Y. Cai, C. Liu, Z. Yu, H. Wu, Y. Wang, W. Ma, Q. Zhang, X. Jia, A flexible and highly conductive quasi-solid single-ion polymer electrolyte for high performance Li-metal batteries. *J. Power Sources* **537**, 231478 (2022).

49. Z. Zeng, X. Chen, M. Sun, Z. Jiang, W. Hu, C. Yu, S. Cheng, J. Xie, Nanophase-separated, elastic epoxy composite thin film as an electrolyte for stable lithium metal batteries. *Nano Lett.* **21**, 3611–3618 (2021).

50. L. Ding, Y. Tan, G. Li, K. Zhang, X. Wang, A healable quasi-solid polymer electrolyte with balanced toughness and ionic conductivity. *Chem. A Eur. J.* **30**, e202400584 (2024).

51. L. Porcarelli, C. Gerbaldi, F. Bella, J. R. Nair, Super soft all-ethylene oxide polymer electrolyte for safe all-solid lithium batteries. *Sci. Rep.* **6**, 19892 (2016).

52. J. Lopez, Y. Sun, D. G. Mackanic, M. Lee, A. M. Foudeh, M. Song, Y. Cui, Z. Bao, A dual-crosslinking design for resilient lithium-ion conductors. *Adv. Mater.* **30**, 1804142 (2018).

53. S. Chen, Y. Zhao, J. Yang, L. Yao, X. Xu, Hybrid solid electrolytes with excellent electrochemical properties and their applications in all-solid-state cells. *Ionics* **23**, 2603–2611 (2017).

54. S. O. Tung, S. Ho, M. Yang, R. Zhang, N. A. Kotov, A dendrite-suppressing composite ion conductor from aramid nanofibres. *Nat. Commun.* **6**, 6152 (2015).

55. W. Liu, J. Chen, Z. Chen, K. Liu, G. Zhou, Y. Sun, M. Song, Z. Bao, Y. Cui, Stretchable lithium-ion batteries enabled by device-scaled wavy structure and elastic-sticky separator. *Adv. Energy Mater.* **7**, 1701076 (2017).

56. R. He, M. Echeverri, D. Ward, Y. Zhu, T. Kyu, Highly conductive solvent-free polymer electrolyte membrane for lithium-ion batteries: Effect of prepolymer molecular weight. *J. Membr. Sci.* **498**, 208–217 (2016).

57. J. Cannarella, X. Liu, C. Leng, P. D. Sinko, G. Y. Gor, C. B. Arnold, Mechanical properties of a battery separator under compression and tension. *J. Electrochem. Soc.* **161**, F3117–F3122 (2014).

58. X. Wei, Y. Deng, X. Hu, Z. Yang, G. Han, H. Xu, Z. Zhang, Thermal stability of PVDF-HFP based gel electrolyte for high performance and safe lithium metal batteries. *Chem. Eng. J.* **502,** 157725 (2024).

59. C. Xian, S. Zhang, P. Liu, L. Huang, X. He, S. Shen, F. Cao, X. Liang, C. Wang, W. Wan, Y. Zhang, X. Liu, Y. Zhong, Y. Xia, M. Chen, W. Zhang, X. Xia, J. Tu, An advanced gel polymer electrolyte for solid-state lithium metal batteries. *Small* **20**, 2306381 (2024).

60. S. Zhang, Z. Li, Y. Zhang, X. Wang, P. Dong, S. Lei, W. Zeng, J. Wang, X. Liao, X. Chen, D. Li, S. Mu, Moderate $Li^+$-solvent binding for gel polymer electrolytes with stable cycling toward lithium metal batteries. *Energy Environ. Sci.* **18**, 3807–3816 (2025).

61. Z. Liu, M. Xi, R. Sheng, W. Wang, J. Ding, Z. Tan, Y. Huang, In situ gelation polymer electrolyte for dendrite-free lithium metal batteries by lewis acid-base interaction. *ACS Mater. Lett.* **7**, 560–565 (2025).

62. J. Gou, Z. Zhang, S. Wang, J. Huang, K. Cui, H. Wang, An ultrahigh modulus gel electrolytes reforming the growing pattern of Li dendrites for interfacially stable lithium-metal batteries. *Adv. Mater.* **36**, e2309677 (2024).

63. Q. Wang, Y. Ma, Y. Wang, X. He, D. Zhang, Z. Li, H. Sun, Q. Sun, B. Wang, L. Fan, In situ catalytic polymerization of $LiNO_3$-containing PDOL electrolytes for high-energy quasi-solid-state lithium metal batteries. *Chem. Eng. J.* **484**, 149757 (2024).

64. Y. Zhang, Z. Li, S. Zhang, J. Li, S. Lei, P. Dong, W. Zeng, J. Wang, X. Chen, D. Li, S. Mu, High-elastic flame-retardant polyacrylate-based gel polymer electrolyte by dual-phase fluorination for highly stable lithium-metal batteries. *Nano Lett.* **25**, 4930–4938 (2025).

65. M. Long, G. Wu, X. Wang, Y. Wang, Self-adaptable gel polymer electrolytes enable high-performance and all-round safety lithium ion batteries. *Energy Storage Mater.* **53**, 62–71 (2022).

66. P. Wang, H. Li, N. Li, J. Sun, F. Xu, X. Tian, X. Shi, Electrospinning fiber membrane-derived gel polymer electrolytes with high mechanical strength and low swelling effect for high-safety lithium metal batteries. *Adv. Funct. Mater.* **35**, 2413544 (2025).

67. D. Chen, M. Zhu, P. Kang, T. Zhu, H. Yuan, J. Lan, X. Yang, G. Sui, Self-enhancing gel polymer electrolyte by in situ construction for enabling safe lithium metal battery. *Adv. Sci.* **9**, e2103663 (2022).

68. J. Huang, Z. Shen, J. Li, A. N. Alodhayb, C. Li, Y. Sun, F. Cheng, Z. Shi, Molecular-level designed gel polymer electrolyte with ultrahigh lithium transference number for high-performance lithium metal batteries. *Chem. Eng. J.* **504**, 158671 (2025).

69. Y. Jiang, S. Zhao, X. Xiao, J. Pi, Y. Wang, N. Yi, L. Zou, Z. Xu, Y. Xiao, X. Ao, G. Ding, W. Zhou, N. Zhou, Z. Xue, Poly(benzoxazine)-based gel polymer electrolytes for lithium metal batteries with ultralong lifespans. *Angew. Chem. Int. Ed. Engl.* **64**, e202510997 (2025).
